# Rational Coformer Selection in the Development of 6-Propyl-2-thiouracil Pharmaceutical Cocrystals

**DOI:** 10.3390/ph16030370

**Published:** 2023-02-28

**Authors:** Francisco Javier Acebedo-Martínez, Carolina Alarcón-Payer, Cristóbal Verdugo-Escamilla, Jesús Martín, Antonio Frontera, Alicia Domínguez-Martín, Jaime Gómez-Morales, Duane Choquesillo-Lazarte

**Affiliations:** 1 Laboratorio de Estudios Cristalográficos, IACT, CSIC-Universidad de Granada, Avda. de las Palmeras 4, 18100 Armilla, Spain; 2 Servicio de Farmacia, Hospital Universitario Virgen de las Nieves, 18014 Granada, Spain; 3 Fundación MEDINA, Centro de Excelencia en Investigación de Medicamentos Innovadores en Andalucía, Parque Tecnológico Ciencias de la Salud, Avda. del Conocimiento 34, 18016 Armilla, Spain; 4 Department of Chemistry, Universitat de les Illes Balears, Crta de Valldemossa km 7.5, 07122 Palma de Mallorca (Baleares), Spain; 5 Department of Inorganic Chemistry, Faculty of Pharmacy, University of Granada, 18071 Granada, Spain

**Keywords:** propylthiouracil, polyphenols, cocrystals, mechanochemistry, crystal engineering

## Abstract

Pharmaceutical multicomponent solids have proved to efficiently modulate the physicochemical properties of active pharmaceutical ingredients. In this context, polyphenols are interesting coformers for designing pharmaceutical cocrystals due to their wide safety profile and interesting antioxidant properties. The novel 6-propyl-2-thiouracil multicomponent solids have been obtained by mechanochemical synthesis and fully characterized by powder and single-crystal X-ray diffraction methods. The analysis of supramolecular synthons has been further performed with computational methods, with both results revealing a robust supramolecular organization influenced by the different positions of the hydroxyl groups within the polyphenolic coformers. All novel 6-propyl-2-thiouracil cocrystals show an enhanced solubility profile, but unfortunately, their thermodynamic stability in aqueous media is limited to 24 h.

## 1. Introduction

**Scheme 1:**
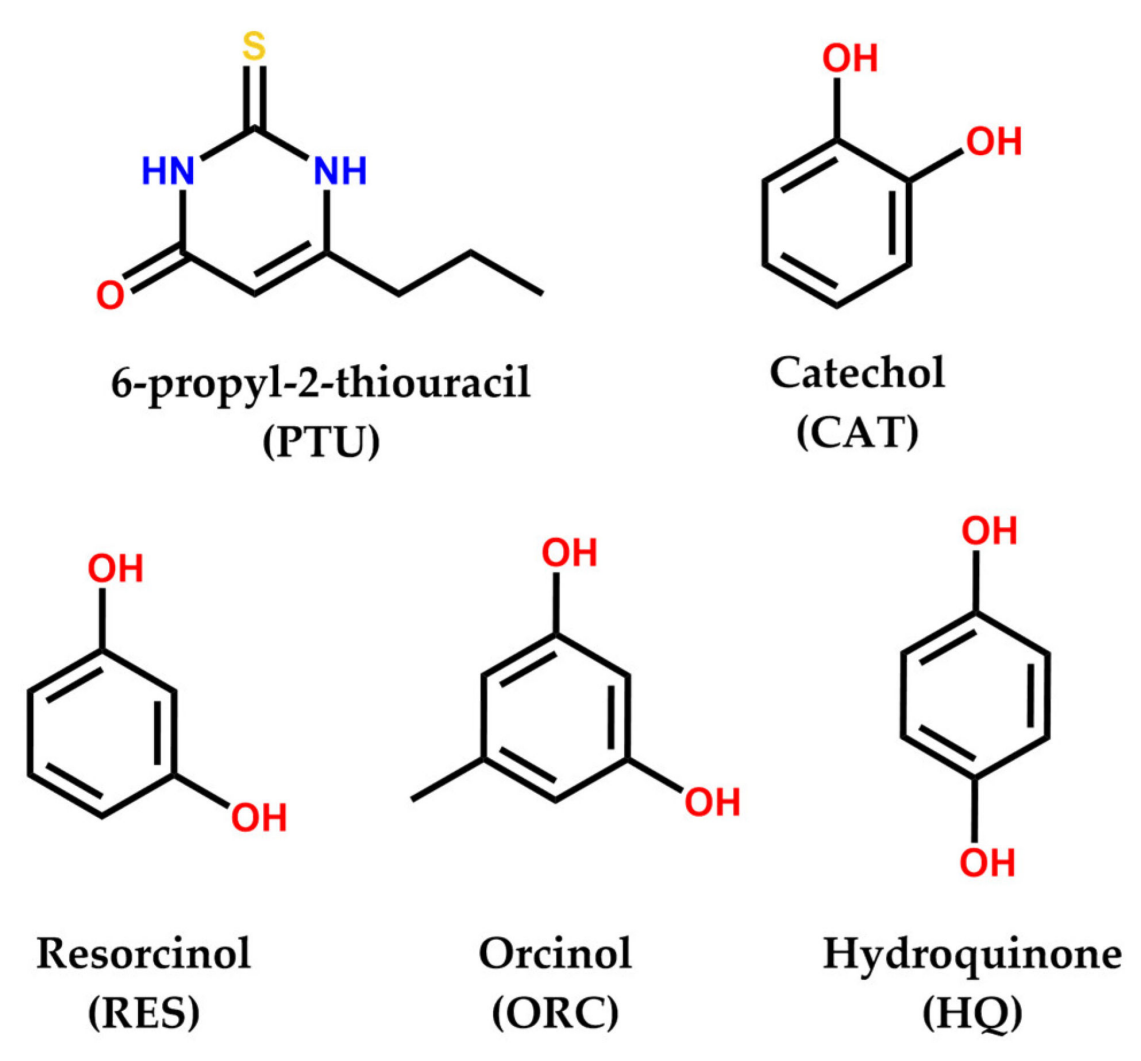
Chemical formula of 6-propyl-2-thiouracil (PTU), catechol (CAT), resorcinol (RES), orcinol (ORC) and hydroquinone (HQ).

6-Propyl-2-thiouracil (PTU, Scheme 1) is a potent human antithyroid drug well-established in the treatment of hyperthyroidism and Graves’ disease [1,2,3]. Its mechanism of action is related to the inhibition of the enzyme thyroperoxidase, reducing the amount of thyroid hormones by avoiding iodide-to-iodine conversion [4]. More recently, a second mechanism of PTU has been provided regarding the peripheral inhibition of enzyme Type I 5’-deiodinase, thus preventing the conversion of thyroxine (T4) to triiodothyronine (T3) [5]. As a result, thyroid hormone activity is significantly reduced, hence forcing the hyperfunction of the thyroid gland. 

PTU primarily undergoes metabolism in the liver. Unfortunately, the use of PTU has been related to acute to severe liver injury, especially relevant in the pediatric population [6,7,8]. This fact has moved PTU to the second line of treatment. However, it is still widely prescribed in patients who do not respond to other treatments, such as methimazole, as an alternative to surgery or radioactive iodine therapy, and during the first trimester of pregnancy and lactation [2,9,10,11].

PTU is hardly soluble in aqueous media. For instance, its solubility in water has been reported at 1.1 mg/mL at 20 °C and 10 mg/mL in boiling water [12], hence limiting its oral bioavailability in gastrointestinal fluids and making it dependent on novel formulations to overcome such limitations. It should be noted that the sulfur atom in its structure (Scheme 1) is susceptible to metabolic oxidation, and its thermal decomposition is known to emit rather toxic fumes of sulfur and nitrogen oxides [13]. Therefore, pharmaceutical formulations of PTU should prevent oxidative events in vivo. 

Pharmaceutical cocrystals, among other multicomponent materials, have thus far proved to be an effective strategy for modulating the physicochemical properties of active pharmaceutical ingredients (APIs) [14]. Such achievements have been accomplished by the application of crystal engineering tools to drug formulation, in which the understanding of the nature, strength, and preferences of intermolecular interactions between molecules, so-called supramolecular synthons, are of paramount importance for appropriate coformer selection [15,16].

Polyphenols are naturally occurring micronutrients mainly found in plants. Their solubility strongly depends on their chemical structure; however, they all exert strong antioxidant power [17]. Evidence on the prevention of diseases by polyphenols has raised the interest of food and pharmaceutical industries in these molecules [17,18,19]. In fact, they are included in the “Substances Added to Food” list [20] and have already been used as coformers in the design of multicomponent pharmaceutical solids with different APIs due to their ability to form O-H···O and O-H···N H-bonds, improving their physicochemical properties [21,22,23,24,25,26].

The aim of this work is to synthesize and characterize novel multicomponent pharmaceutical solids containing the antithyroid drug PTU and different polyphenols as coformers. Our purpose is to assess whether the relative position of hydroxyl groups present in the selected polyphenols isomers (catechol, resorcinol, orcinol and hydroquinone, see Scheme 1) affects H-bonding formation and, consequently, the crystal architecture of the novel pharmaceutical cocrystals. Herein, molecular recognition is thoroughly characterized by single-crystal X-ray diffraction and computational methods. In addition, the influence of such structural changes on the physicochemical properties of the novel multicomponent solids has been evaluated. In particular, thermal stability, thermodynamic stability in aqueous media, as well as solubility studies have also been performed to evaluate the effectiveness of the novel multicomponent formulation. 

## 2. Results and Discussion

### 2.1. Cocrystals Containing PTU

A CSD survey (version 5.43, November 2022) revealed 17 structures, including 3 solvates [3,27], 7 cocrystals [25,27,28], 1 solvated molecular salt [29], and 5 metal complexes [30,31]. The structural analysis of the multicomponent crystals of PTU (excluding metal complexes) listed in the CSD has shown two main structural features: (i) infinite chains of PTU molecules built by hydrogen bonding interactions, as observed in the crystal structure of PTU [28,32], that are further connected by H-bonds involving coformer molecules and (ii) pairs of hydrogen-bonded PTU molecules surrounded and connected by coformer molecules. One exception in this classification is observed in the crystal structure of the molecular salt of PTU with 2,4-di-amino-pyrimidine where a pair of PTU molecules is associated by π,π-stacking interactions and further reinforced by H-bonds involving water molecules [29].

### 2.2. Virtual Cocrystal Screening

A virtual screening was conducted in order to evaluate the propensity of the selected polyphenolic coformer candidates to form cocrystals. The COSMOquick software was used to validate our selection. This tool calculates the excess enthalpy of formation (H_ex_) between PTU and the corresponding coformer in a supercooled liquid phase [33]. A list of SMILES of the molecules was used as input data. Table 1 shows COSMOQuick calculations for our list of candidates, including other coformer molecules involved in the formation of cocrystals/salts reported in the CSD survey. Those compounds with negative H_ex_ values show an increased probability of forming cocrystals since H_ex_ is a rough approximation of the free energy of cocrystal formation ΔG_cocrystal_. The results confirm PTU’s preference to form cocrystals with polyphenolic coformers.

### 2.3. Cocrystal Synthesis

The mechanochemical synthesis was conducted by liquid-assisted grinding (LAG). This technique allows the synthesis of new multicomponent materials in a quick and efficient way without consuming high amounts of time and organic solvents. 

For LAG reactions, mixtures of PTU, the respective coformers, and 150 µL of dichloromethane as a liquid additive were placed in stainless steel jars along with 2 stainless steel balls of 7 mm diameter. After 30 min of ball-milling, the samples were characterized by powder X-ray diffraction (PXRD) and compared with the patterns of the initial reagents to determine the formation of new materials.

**Figure 1:**
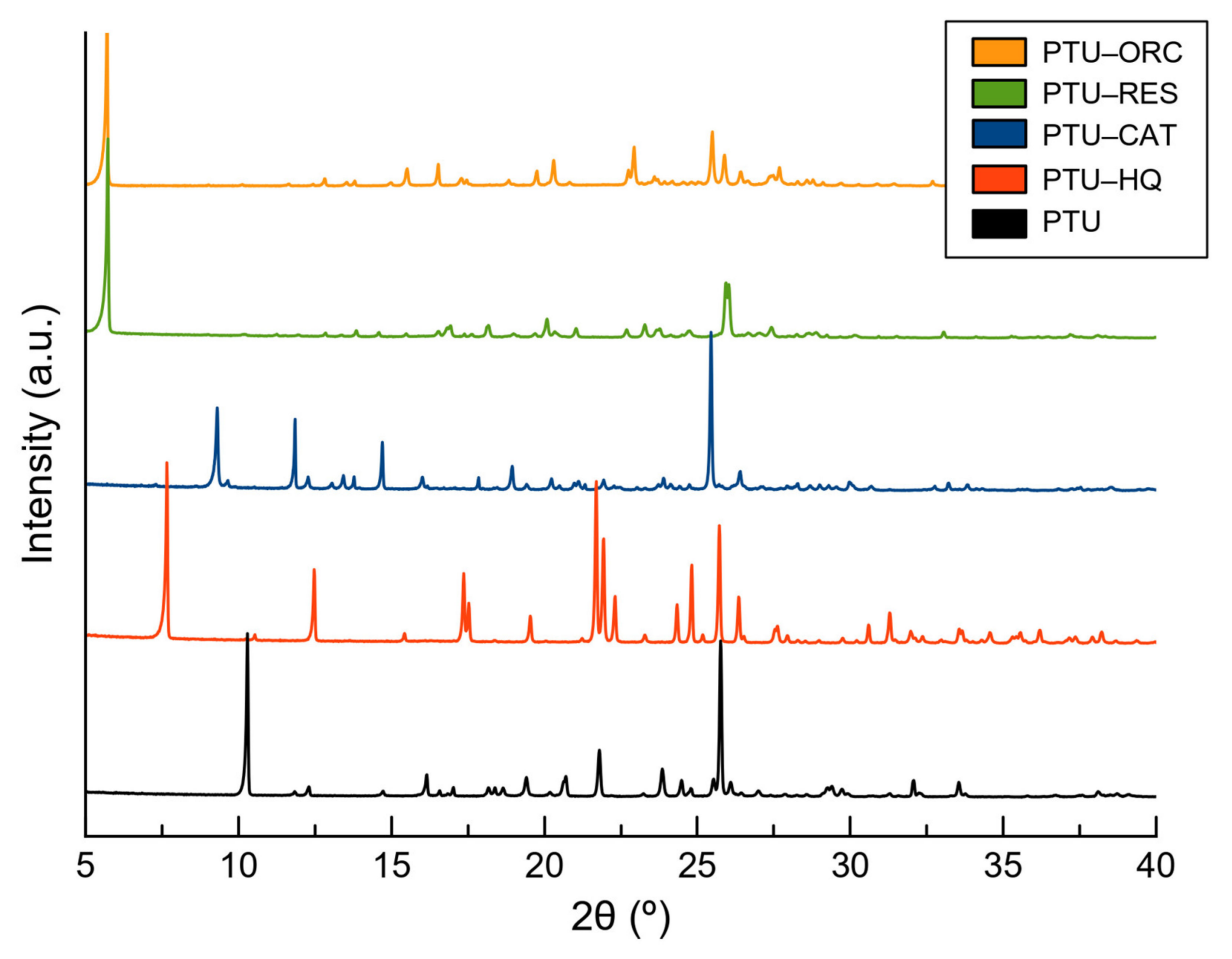
Powder X-ray diffraction (PXRD) patterns of PTU and the new phases obtained by LAG.

The above-mentioned reactions lead to the formation of four new materials. However, all of them, with the exception of PTU–CAT, appeared with an excess of the coformer molecule (Figure S1, in Supplementary Materials), indicating the formation of novel phases but with a different stoichiometry from the one initially used. At this point, PTU–RES, PTU–HQ, and PTU–ORC reactions were performed using a 2:1 stoichiometry, finally leading to the formation of the new pure phases with no additional peak from parent reagents, as shown in Figure 1.

For further determination of the crystalline structure by single-crystal X-ray diffraction (SCXRD), saturated solutions were prepared using the product of the LAG and different organic solvents. After two days of slow solvent evaporation at room temperature, crystals of PTU–RES and PTU–ORC with good quality for SCXRD appeared in methanol, while crystals of PTU–CAT and PTU–HQ appeared in dichloromethane.

After crystal structure determination, simulated powder patterns of the new phases were obtained and compared with the PXRD patterns obtained by LAG (Figure S2). The good agreement of the simulated and experimental PXRD patterns confirmed the phase purity of the bulk product obtained by mechanochemistry.

### 2.4. Crystal Structure Analysis

**Figure 2:**
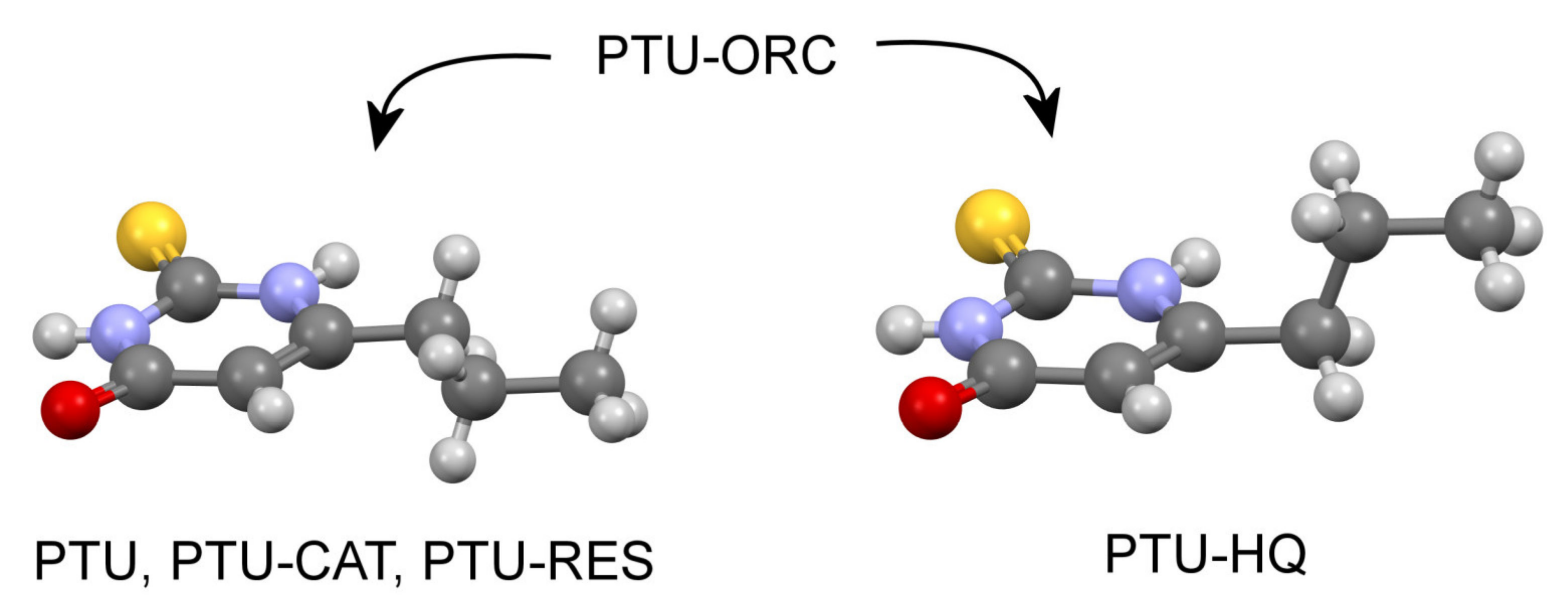
Schematic representation of the conformation of the side chain of PTU in the cocrystals reported in this work and in the parent PTU molecule.

Crystallographic data for the reported cocrystals are summarized in Table S1. Asymmetric units are represented in Figure S3. Hydrogen bonds and other non-covalent interactions information are presented in Tables S2 and S3. In the reported cocrystals, PTU exhibits different side-chain conformations. While the PTU structure reported in the literature has an extended conformation [28,32], the dihedral angle between the plane of the thiouracil ring and the plane of the side chain ranges from 3.81 to 88.18 degrees in the novel phases, with an extended arrangement being preferred in the cocrystals with CAT and RES as coformers and with a bent conformation in PTU–HQ. Interestingly, PTU–ORC exhibits PTU in the two above-referred conformations (Figure 2).

The different position of the substituents in the isomers used as coformers has an effect on the supramolecular organization in the studied compounds, as detailed below.

**Figure 3:**
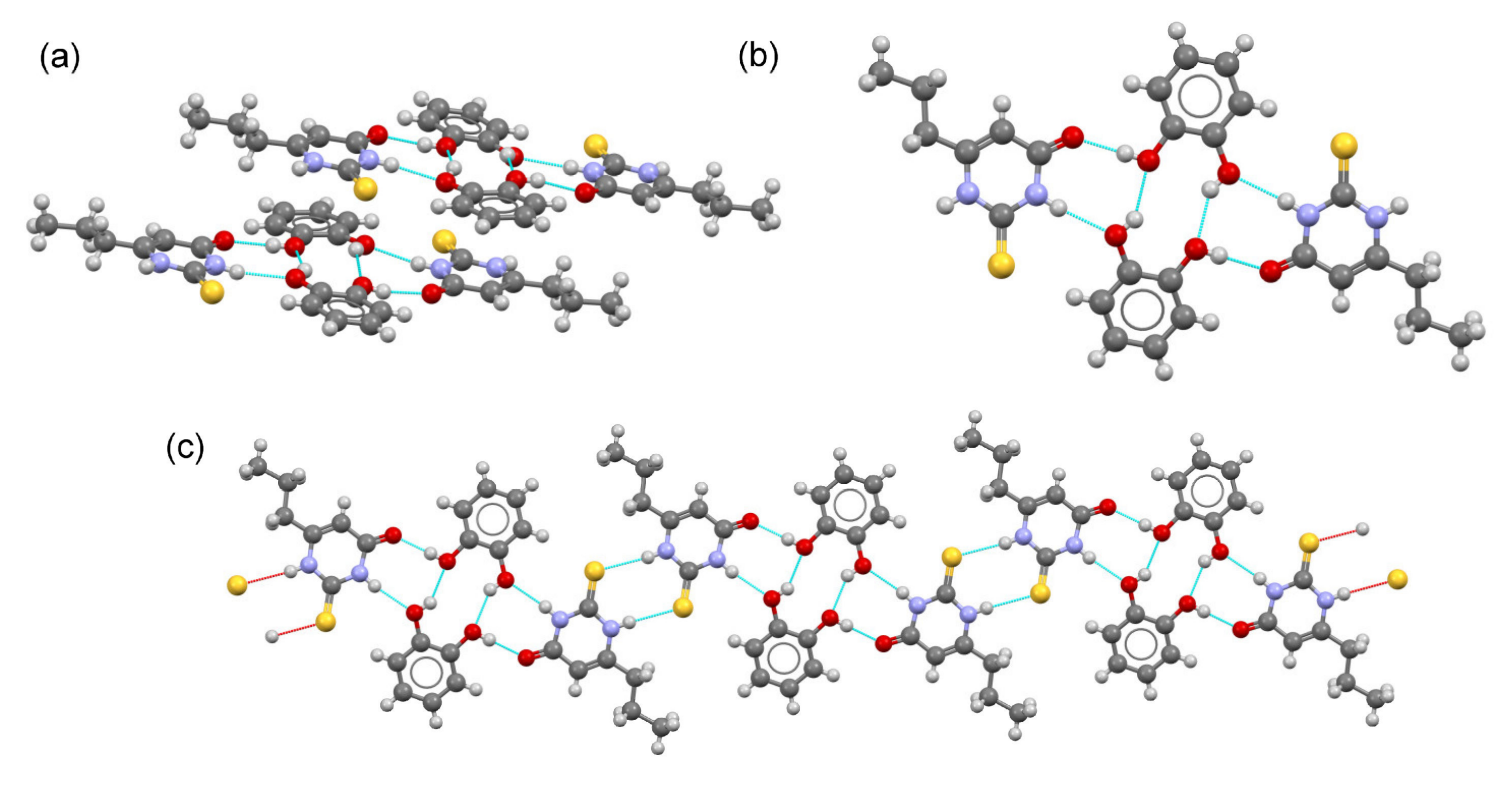
(**a**) Asymmetric unit in the cocrystal PTU–CAT. (**b**) Detailed view of the tetrameric structure. (**c**) Fragment of the ribbon structure in PTU–CAT.

**Figure 4:**
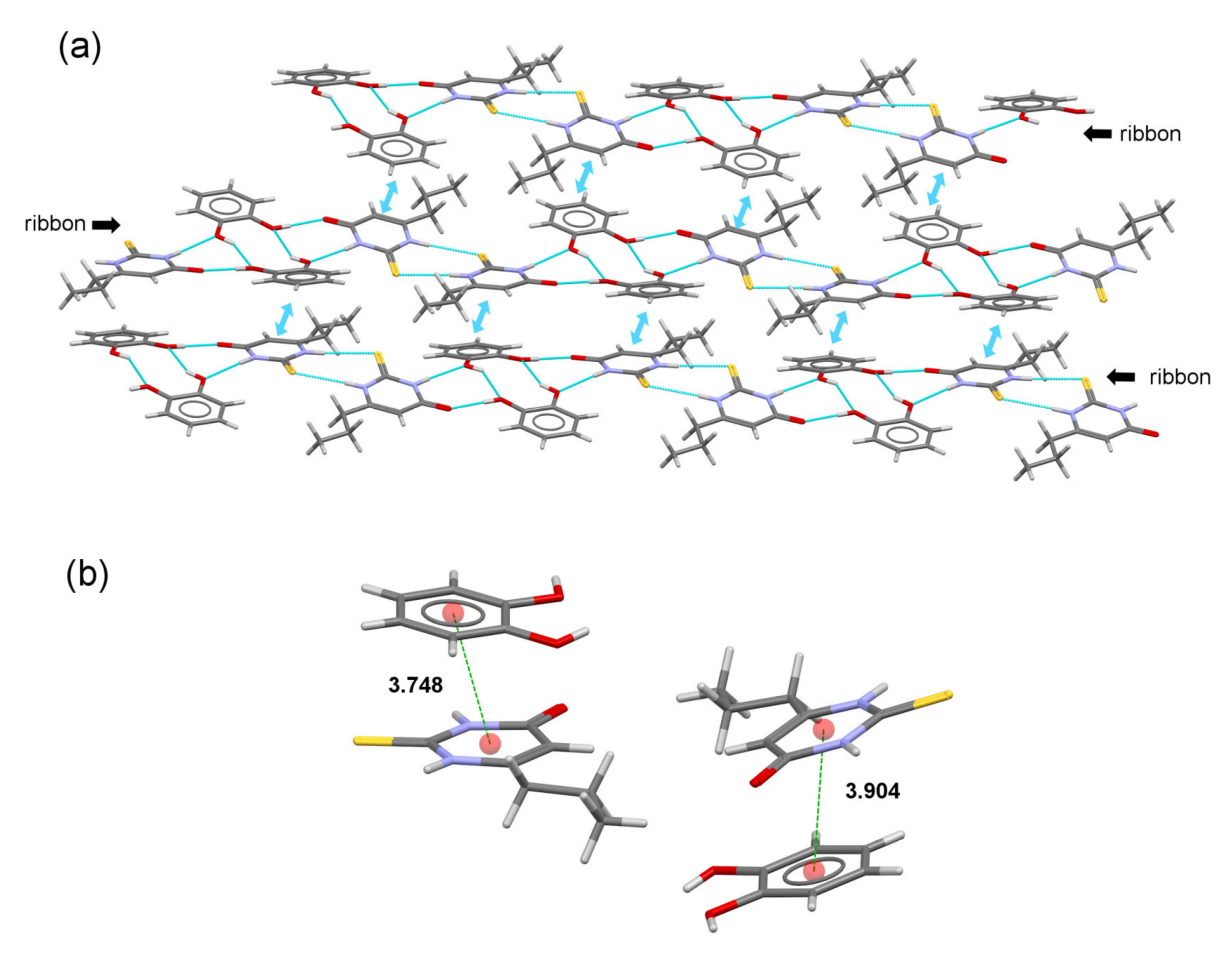
(**a**) Fragment of the 2D-layered structure built by association of ribbons by π,π-stacking interactions. (**b**) Details of the π,π-stacking interactions between PTU and CAT molecules (distances in Å).

PTU–CAT cocrystal crystallized in the monoclinic space group P2_1_, with an asymmetric unit containing four molecules of PTU and four molecules of CAT, the latter showing intramolecular hydrogen bonding. PTU:CAT pairs associate through H-bonding interactions to form a tetramer (hydrogen-bonded $R_{2}^{2}\left(10\right)$ motif between two CAT molecules and two $D_{1}^{1}\left(2\right)$ motifs between PTU and CAT molecules, Figure 3b). Additional H-bonds involving PTU molecules (centrosymmetric -N-H···S hydrogen bonds, $R_{2}^{2}\left(8\right)$ motif) associate with tetramers to build a ribbon structure extending in the [1 0 −1] direction (Figure 3c). In the tetramer, PTU dimer formation is disrupted by the insertion of two CAT molecules. π,π-stacking interactions between pairs of PTU and CAT molecules further associate with ribbons to generate a 2D layered structure (Figure 4a). Finally, these layers are then assembled by -C-H (methylene, PTU)···π (catechol) interactions to build a 3D structure. 

**Figure 5:**
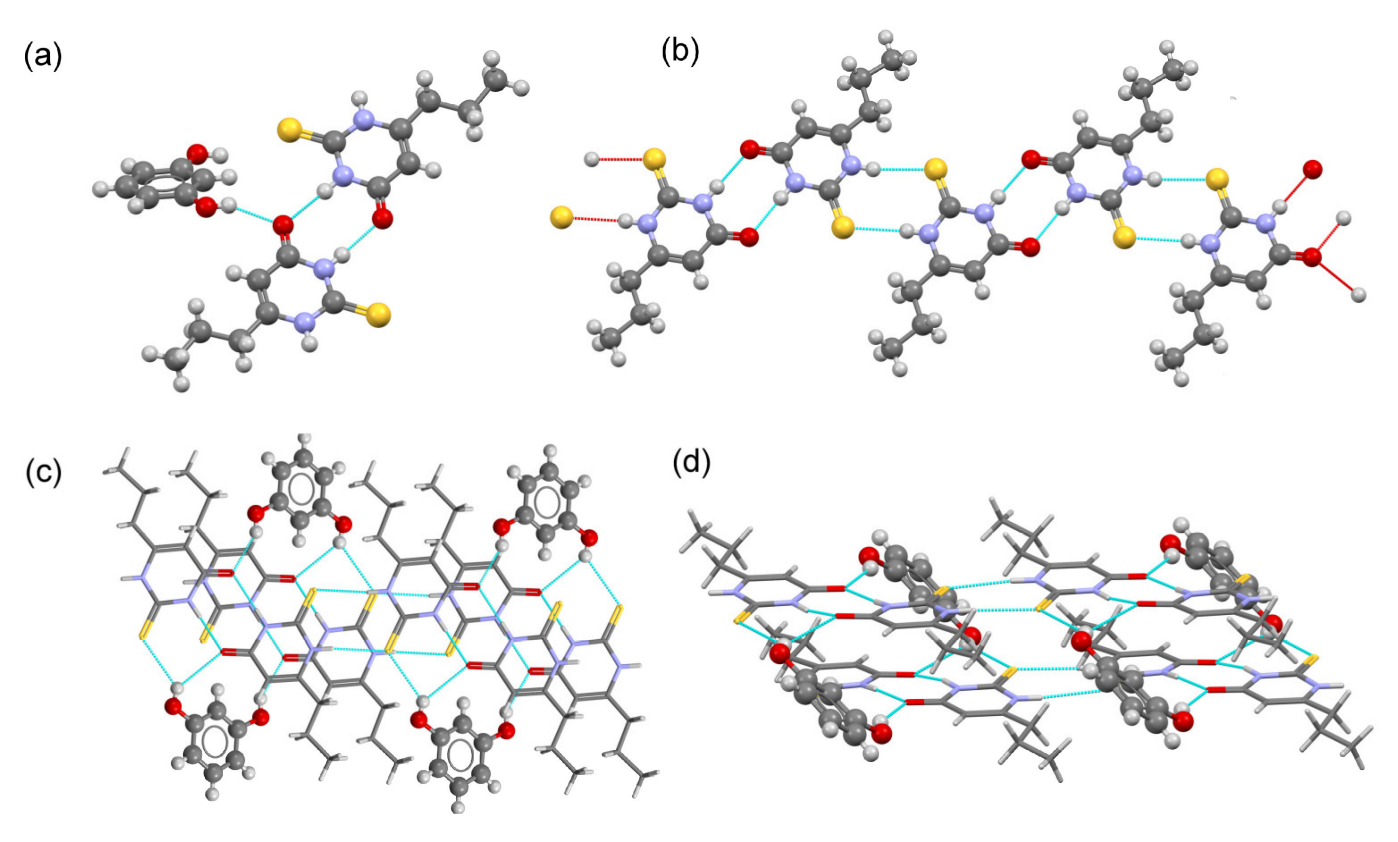
(**a**) Asymmetric unit in cocrystal PTU–RES. (**b**) Detailed view of the PTU chain generated by H-bonding interactions. (**c**) Fragment of a belt-type structure built by connecting two PTU chains through H-bond involving RES molecules. (**d**) Lateral view of the belt-type structure in PTU–RES.

The PTU–RES cocrystal crystallized in the triclinic P-1 space group. The asymmetric unit is composed of PTU and RES in a 2:1 stoichiometric ratio. PTU molecules associate by H-bonding interactions, $R_{2}^{2}\left(8\right)$ homosynthons (N1-H1···S12, N11-H11···S2, and N3-H3···O14, N13-H13···O4) to generate an infinite chain extending along the c-axis. RES molecules, in syn-syn conformation, reinforce the chain by H-bonding interactions involving one of their O atoms and the carbonyl group of PTU, O20-H20···O14 (carbonyl, PTU). The remaining -OH group connects with an adjacent chain through H-bonds between OH and S12 to build a belt-type structure where -C=S···π(resorcinol) interactions contribute to its cohesion. -C-H (methylene, PTU)···π (resorcinol) interactions associate with belts to generate a 2D layer exposing the PTU aliphatic groups to the periphery. The 3D supramolecular structure is then obtained by stacking 2D layers facing their hydrophobic groups (Figure 5).

**Figure 6:**
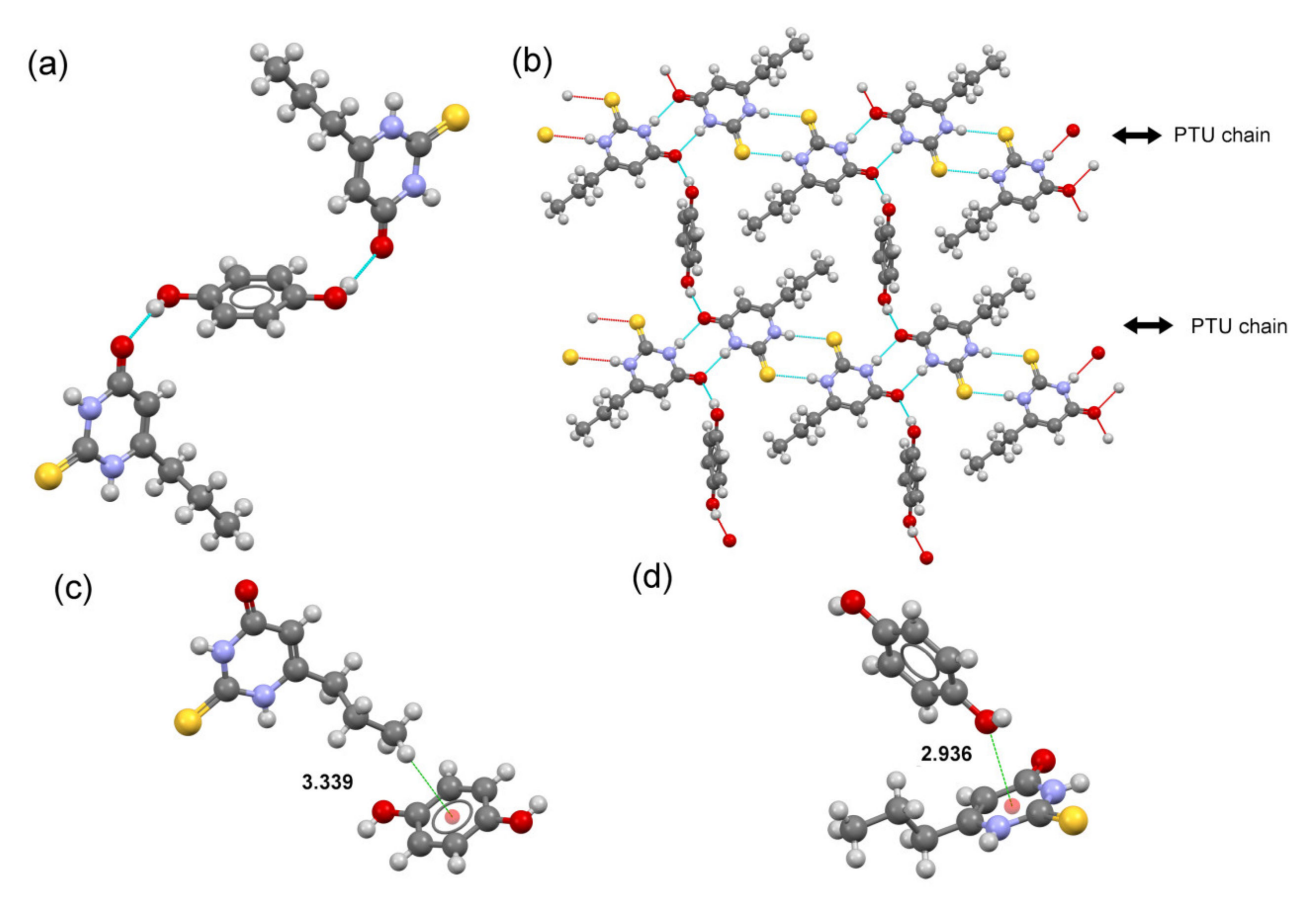
(**a**) Asymmetric unit in cocrystal PTU–HQ. HQ molecule is located in an inversion center. (**b**) Fragment of the 2D layered structure generated by H-bonds and C-H··· π interactions. (**c**) Detailed view of the C-H··· π interaction between PTU and CAT molecules (distance in Å). (**d**) Detailed view of the lone pair···π interaction between CAT and PTU molecules that connects layers to give a 3D structure (distance in Å).

PTU–HQ crystallized as a cocrystal in the triclinic P-1 spacegroup. The asymmetric unit consists of one molecule of PTU and half a molecule of HQ located in an inversion center, giving a 2:1 stoichiometric ratio (Figure 6a). As in the previously described structure, PTU molecules associate to build a hydrogen-bonded infinite chain using the same $R_{2}^{2}\left(8\right)$ homosynthons, but in PTU-HQ, the role of the HQ coformer is different, connecting chains directly by H-bonding interactions to generate a 2D-layered structure where -CH_3_···π interactions participate in their cohesion (Figure 6b). The 3D architecture is finally obtained by stacking layers through lone pair···π interactions involving both HQ and PTU molecules.

**Figure 7:**
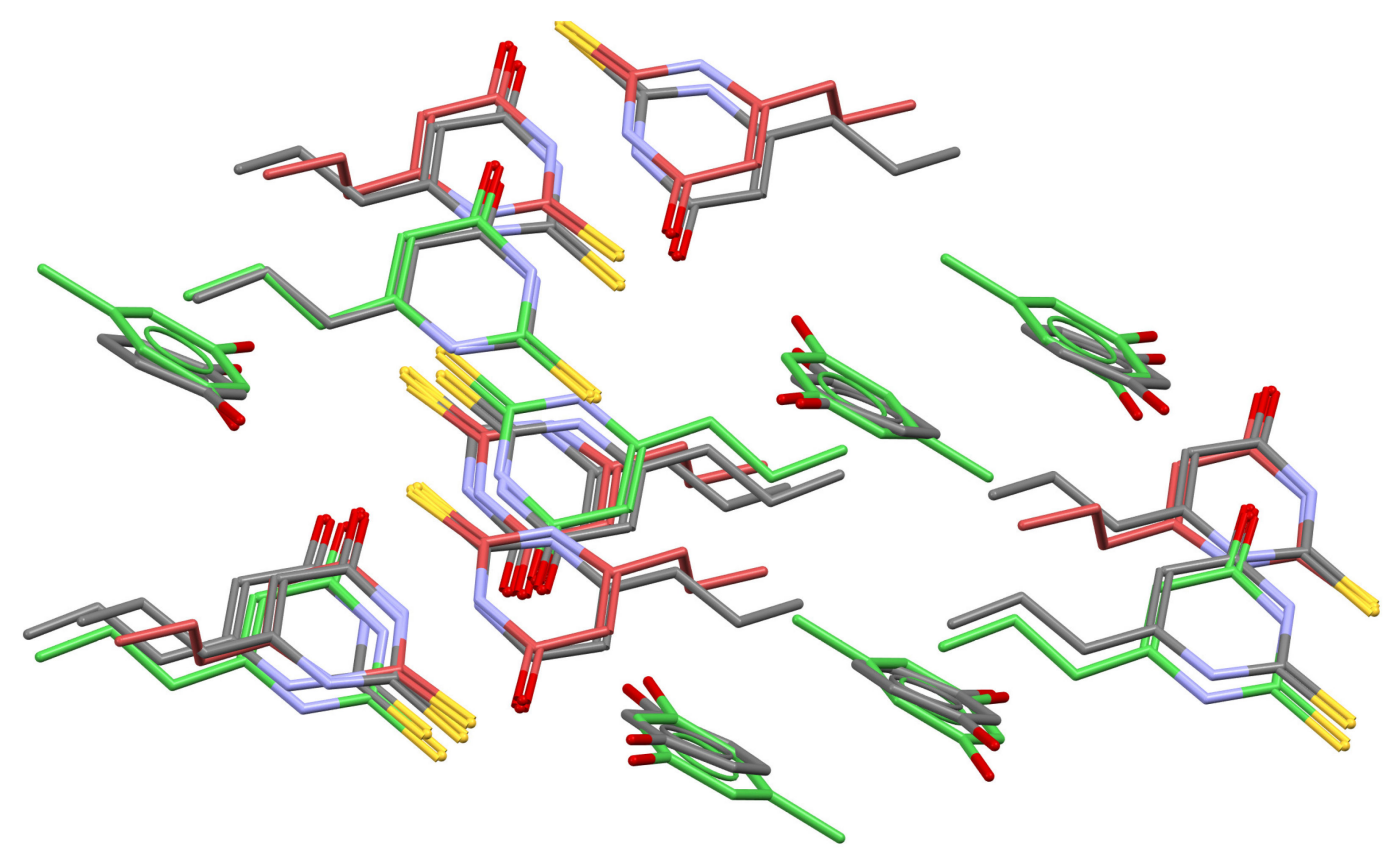
Crystal packing similarity plot comparing the PTU–RES and PTU–ORC crystal structures. Green: matching molecules. Red: not matching molecules due to different conformation of PTU molecules.

The PTU–ORC cocrystal crystallized in the triclinic crystal system with the P-1 space group. The asymmetric unit is composed of PTU and ORC in a 2:1 stoichiometric ratio. Cocrystals of PTU with related RES and ORC coformers are similar. The unit cell similarity index Π (0.012) [34] and the PXRD similarity scores (calculated from the packing similarity tool in Mercury [35]) confirm the isomorphic relationship between both cocrystals (Figure 7). The results obtained from the latter calculations showed that although 9 out of 15 molecules were matched in the pairs of cocrystals, their crystal packing is the same (PXRD similarity: 0.979; RMSD (Å): 0.442). The deviation from total matching is a consequence of the different alkyl chain conformation observed in the two symmetry-independent PTU molecules in the PTU-ORC cocrystal. 

**Figure 8:**
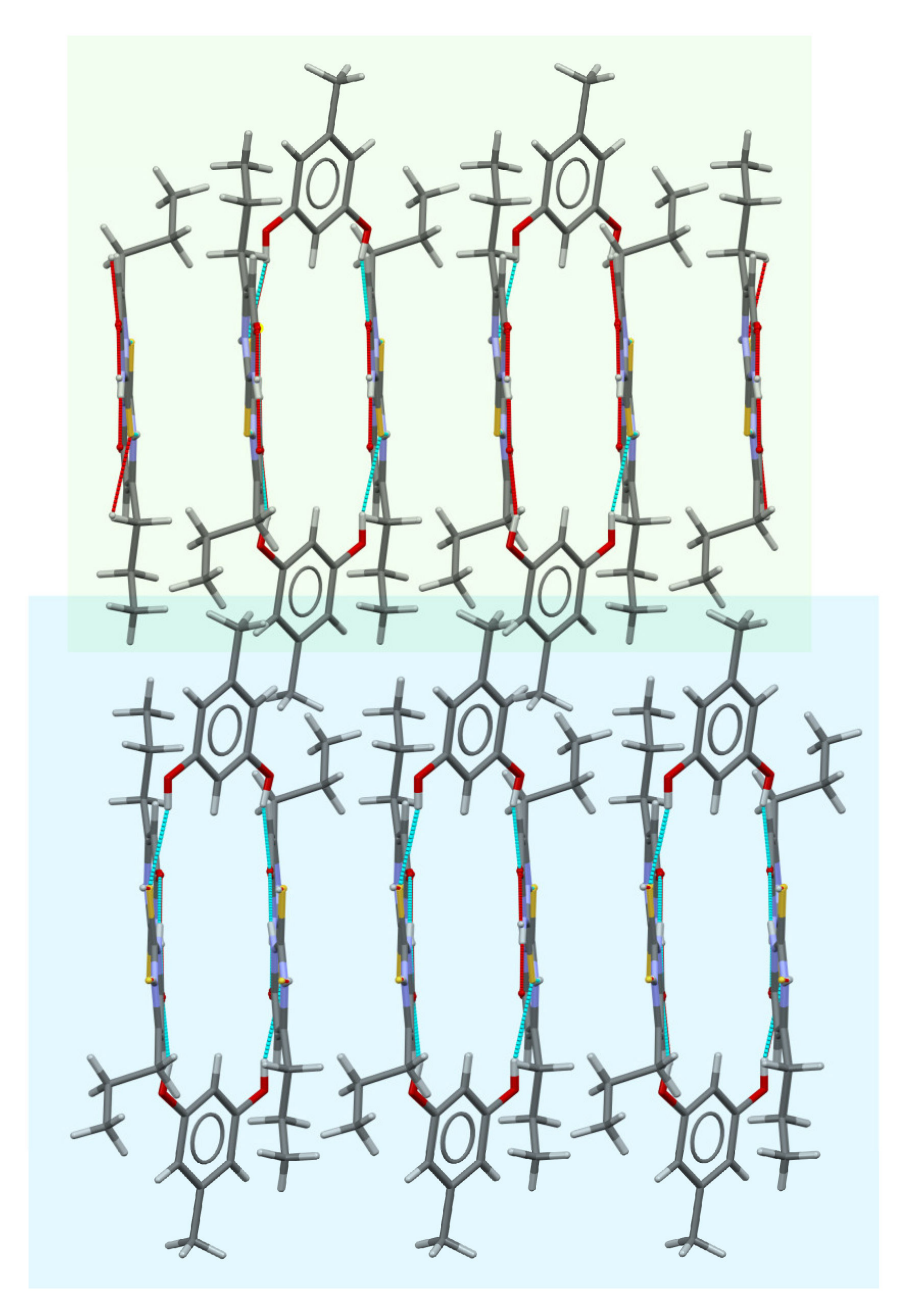
Packing arrangement of 2D layers in the crystal structure of PTU–ORC.

Similarly, as in PTU–RES, pairs of infinite H-bonded PTU chains are connected by ORC coformers to generate belts that are further associated by C-H···π interactions to assemble a layer structure. Stacks of these layers built the 3D supramolecular structure (Figure 8).

While cocrystals with RES and ORC coformers are iso-structural and therefore have a similar supramolecular architecture, the arrangement of hydroxyl substituents in the other cocrystals results, as expected, in different crystal packings. A common feature in all the cocrystal structures in this work is the effective separation of PTU chains that are cohesive in the reported PTU structure by means of hydrogen bonds that expose hydrophobic environments. This feature is more notable in the PTU–CAT cocrystal, where catechol molecules interrupt PTU chains to surround PTU dimers. These findings should have implications on the solid-state physicochemical properties of the cocrystals compared to the parent API, as will be seen later.

### 2.5. DFT Study of Non-Covalent Interactions

**Figure 9:**
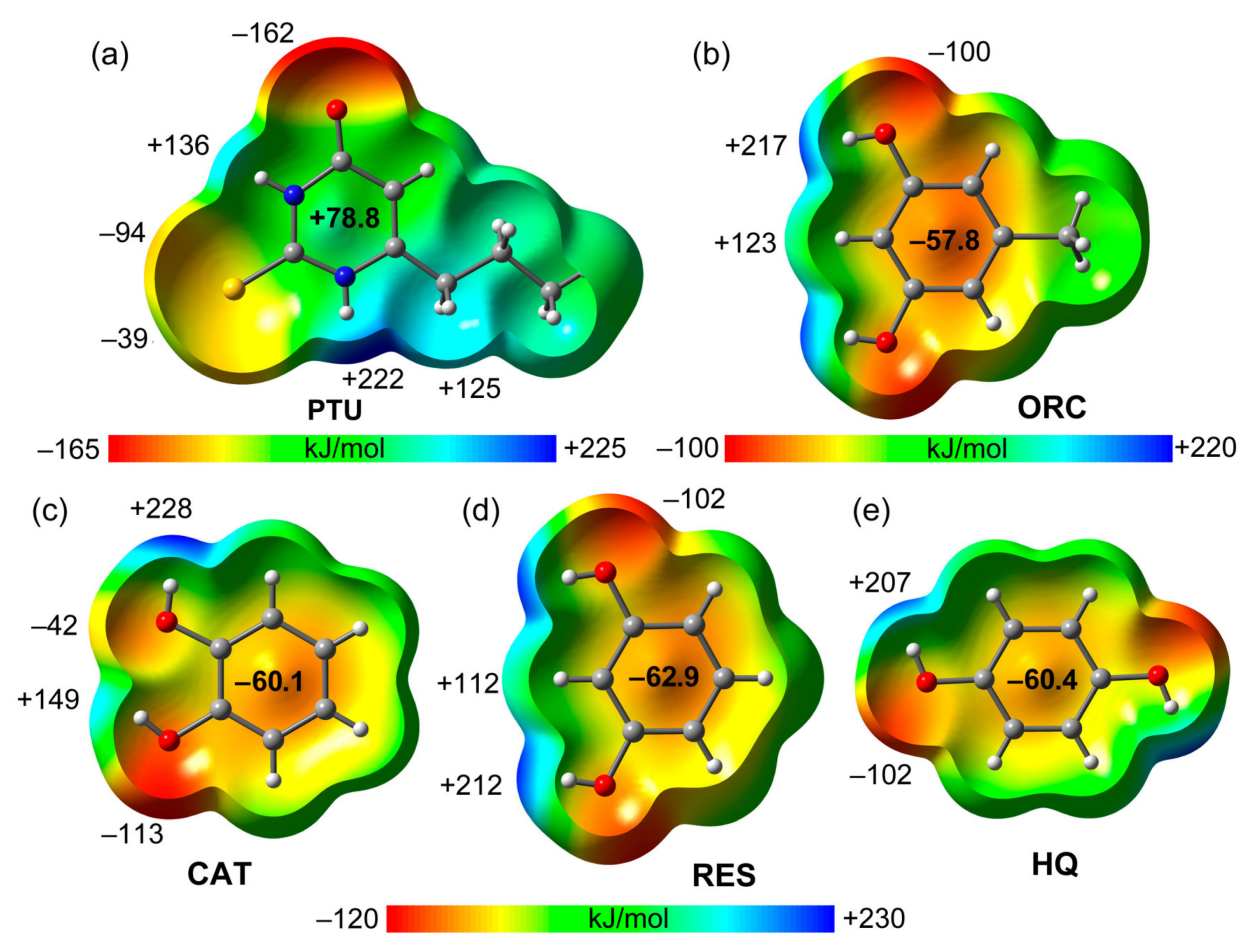
MEP surfaces of the salts of PTU (**a**), ORC (**b**), CAT (**c**), RES (**d**), and HQ (**e**) at the PBE0-D3/def2-TZVP level of theory (density isovalue 0.001 a.u.). The energies are given in kJ/mol.

The theoretical study is mainly devoted to the energetic analysis of the H-bonded synthons described in the structural section. First, we computed the MEP surfaces of the different coformers to explore their relative H-bond donor/acceptor ability. The MEP surfaces are given in Figure 9 for 6-propyl-2-thiouracil (PTU) and the four polyphenols used in this work. For PTU, the MEP minimum is located at the O-atom (–162 kJ/mol) followed by the S-atom (negative belt, –94 kJ/mol). The MEP is significantly more positive on the extension of the C=S bond due to the presence of a σ-hole (–39 kJ/mol). The maximum is located at the N1–H bond (+222 kJ/mol) followed by the N3–H bond (+136 kJ/mol). The MEP values are also large and positive at the CH bonds of the propyl group, especially those closer to the thiouracil ring, likely due to the influence of the adjacent N1–H group. The MEP value over the center of the ring is positive (+78.8 kJ/mol), thus disclosing its ability to interact with electron-rich atoms. Regarding the polyphenol rings, the MEP maxima are located at the -OH groups, as expected, ranging from +207 kJ/mol to +228 kJ/mol. In HQ, RES, and ORC molecules, the MEP values are equivalent at both -OH groups. However, in CAT, the -OH group that points to the adjacent -OH presents a much lower MEP value (+149 kJ/mol). The minima are located at the O-atoms of the -OH groups, ranging from –100 to –113 kJ/mol. Again, for CAT, one of the O-atom presents a much lower (in absolute value) MEP (–42 kJ/mol) due to the presence of the adjacent -OH group. Finally, all polyphenols have a quite π-basic aromatic ring with MEP values ranging from –60.1 to –62.9 kJ/mol. Taken together, the MEP analysis reveals that all coformers are better H-bond donors than acceptors. In addition, the fact that the PTU exhibits greater MEP minimum and maximum anticipates a higher ability to form self-assembled dimers than the rest of the coformers. 

The cocrystals reported herein present recurrent synthons in the solid state that have been analyzed theoretically using the quantum theory of atoms in molecules (QTAIM) combined with the non-covalent interaction plot (NCIplot). The latter is useful to reveal interactions in real space by plotting the reduced density gradient (RDG) isosurfaces. Moreover, a color code is used to denote the attractive nature of the interactions. In this work, green and blue colors are used for weak and strongly attractive interactions. 

**Figure 10:**
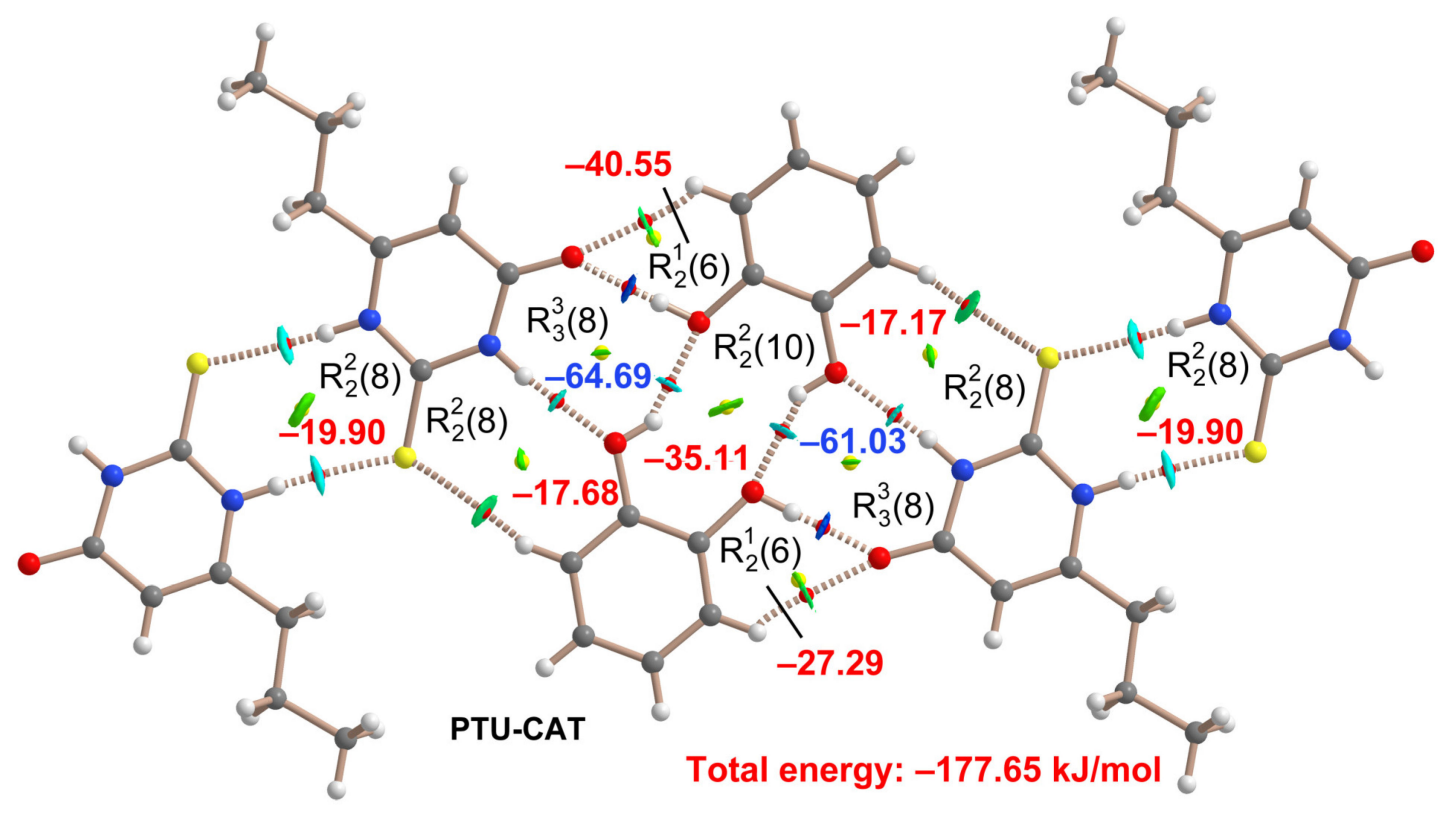
QTAIM/NCIPlot analysis of intermolecular bond and ring CPs (red and yellow spheres, respectively), bond paths, and RDG isosurfaces of the H-bonded assembly of PTU–CAT cocrystal. The individual association energies of each synthon are indicated using a red or blue font inside the supramolecular rings.

For all cocrystals, we have selected fragments where all types of H-bonds observed in the solid state are represented. Our purpose is to study the relative energies of all synthons and their prevalence in the solid state of the different cocrystals. Figure 10 shows the QTAIM/NCIplot analysis of the PTU–CAT fragment (corresponding to one of the asymmetric units), revealing that each H-bond is characterized by a bond critical point (CP, small red spheres) and bond path (dashed bonds) connecting the H to the N-, O-, and S-atoms of the different coformers. PTU self-assembles through two N1–H···S H-bonds, generating a $R_{2}^{2}\left(8\right)$ homosynthon and leaving the O-atom of the thiouracil ring (the most nucleophilic region) free to interact with the coformer. Similarly, CAT molecules also form self-assembled dimers held together by O–H···O H-bond $R_{2}^{2}\left(10\right)$ homosynthons, leaving the most electrophilic part of the molecule (OH) to interact with the coformer. In fact, the OH···O H-bond is the strongest interaction, as revealed by the color of the RDG isosurface that characterizes this H-bond. The O-atom of thiouracil also establishes a CH···O H-bond with an aromatic CH bond, thus generating a $R_{2}^{1}\left(6\right)\;$ synthon. The H-bonding interactions are also revealed by the NCI plot index, showing dark blue reduced density gradient (RDG) isosurfaces for the O–H···O H-bonds, blue for the N–H···O,S H-bonds, and green RDG isosurfaces for the C–H···S,O H-bonds, thus evidencing that the OH···O are the strongest ones. This is further confirmed by the association energies computed for each synthon (values in red or blue in Figure 10, inside each supramolecular ring). It can be observed that the $R_{3}^{3}\left(8\right)$ and $R_{2}^{2}\left(10\right)$ homosynthon and the $R_{2}^{1}\left(6\right)$ heterosynthon are the strongest ones, both involving OH···O H-bonds. The total association energy of the assembly was computed as a sum of the association energies of all synthons with red values (the H-bonds of the synthons with blue energies are already included in the red ones). The total association energy of the assembly is –177.65 kJ/mol, which confirms the important role of the H-bonds in this cocrystal. We have also performed the study for the equivalent assembly using the other asymmetric fragment, obtaining a similar association energy (45.0 kJ/mol). 

**Figure 11:**
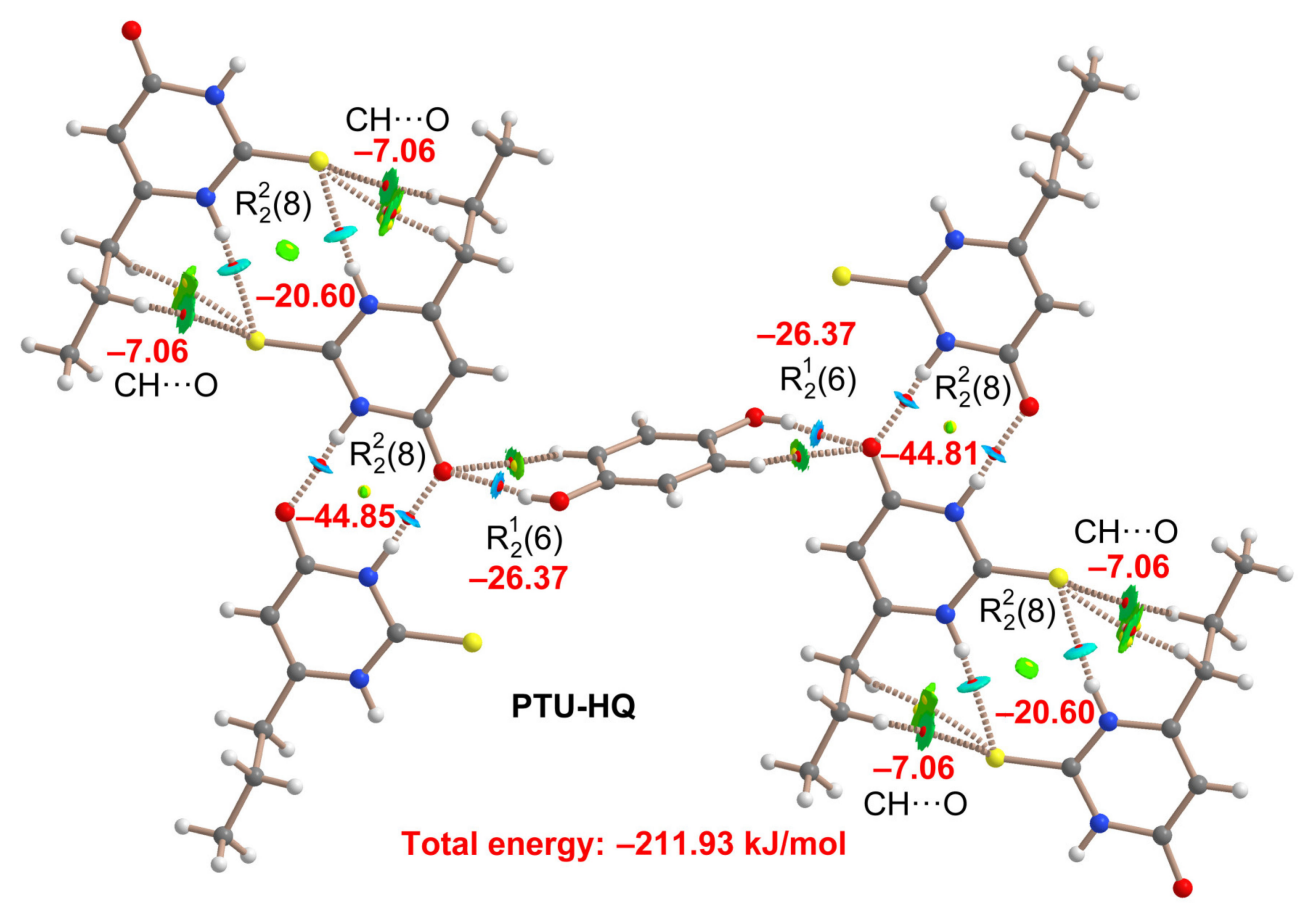
QTAIM/NCIPlot analysis of intermolecular bond and ring CPs (red and yellow spheres, respectively), bond paths, and RDG isosurfaces of the H-bonded assembly of PTU–HQ cocrystal. The individual association energies of each synthon or CH···O H-bond are indicated using a red font inside the supramolecular rings or adjacent to the CH···O H-bonds.

Figure 11 shows the QTAIM/NCIplot analysis of the PTU–HQ fragment. Herein, PTU forms two $R_{2}^{2}\left(8\right)$ centrosymmetric dimers, one through two N–H···S H-bonds, with an association energy of –20.60 kJ/mol (similar to PTU–CAT) and the other one via N–H···O H-bonds with greater association energy (–44.85 kJ/mol). This homotrimer, supported by both types of $R_{2}^{2}\left(8\right)$ synthons, is bridged by the HQ coformer by means of weak CH···O and strong OH···O bonds, generating $R_{2}^{1}\left(6\right)$ supramolecular rings (–26.37 kJ/mol), similar to those described in PTU–CAT. The combined QTAIM and NCIplot analysis also reveals that the $R_{2}^{2}\left(8\right)$ synthon held together by two symmetrically equivalent N–H···S interactions is further stabilized by two pairs of ancillary CH···S contacts involving the aliphatic protons. The energy associated with each pair is –7.06 kJ/mol. The total association energy of the assembly is –211.93 kJ/mol, which is similar to that observed in PTU–CAT.

**Figure 12:**
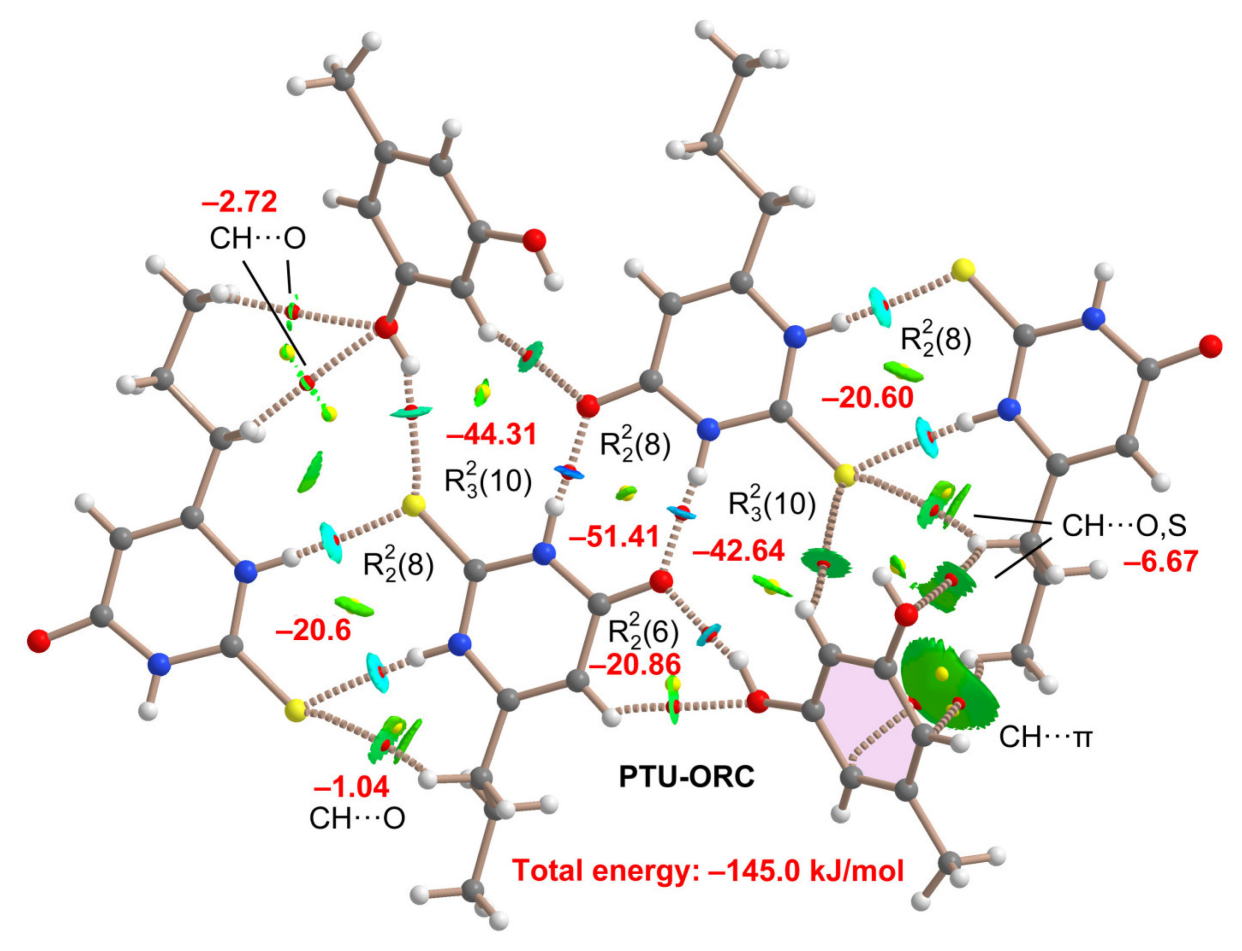
QTAIM/NCIPlot analysis of intermolecular bond and ring CPs (red and yellow spheres, respectively), bond paths, and RDG isosurfaces of the H-bonded assembly of PTU–ORC cocrystal. The individual association energies of each synthon or CH···O H-bond are indicated using a red font inside the supramolecular rings or adjacent to the CH···O H-bonds.

**Figure 13:**
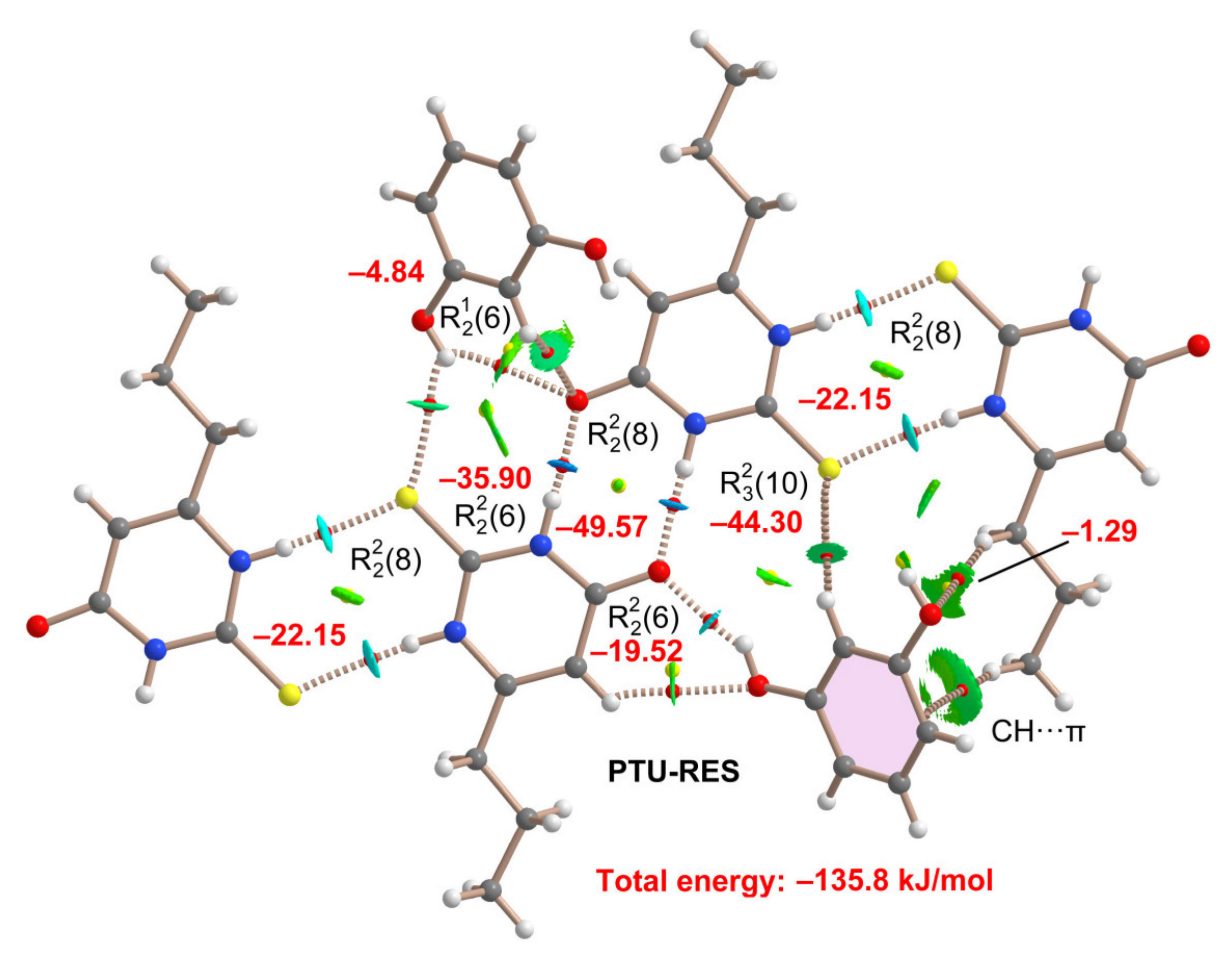
QTAIM/NCIPlot analysis of intermolecular bond and ring CPs (red and yellow spheres, respectively), bond paths, and RDG isosurfaces of the H-bonded assembly of PTU–RES cocrystal. The individual association energies of each synthon or CH···O H-bonds are indicated using a red font inside the supramolecular rings or adjacent to the CH···O H-bonds.

Figure 12 and Figure 13 show the QTAIM/NCIplot analyses of the assemblies corresponding to PTU–ORC and PTU–RES cocrystals, respectively. In both cases, similarly to PTU–HQ, the PTU forms two types of $\;R_{2}^{2}\left(8\right)$ homodimers that extend the PTU into 1D supramolecular polymers. The $R_{2}^{2}\left(8\right)$ homodimer characterized by two N–H···S H-bonds have association energies of –20.60 kJ/mol and –22.15 kJ/mol for PTU–ORC and PTU–RES cocrystals, respectively. The $R_{2}^{2}\left(8\right)$ homodimer characterized by two N–H···O H-bonds have much larger association energies, which are –51.41 kJ/mol and –49.57 kJ/mol for PTU–ORC and PTU–RES, respectively. ORC and RES coformers interact with the infinite 1D chains using two different binding modes that have also been represented in Figure 12 and Figure 13. For ORC (Figure 12), one binding mode implies the formation of OH···S and CH···O H-bonds with two different PTU molecules as a part of a $R_{3}^{2}\left(10\right)$ synthon. In this binding mode, two very weak CH···O interactions are also revealed by the QTAIM/NCIplot analysis (–2.72 kJ/mol). For the other binding mode, ORC also participates in the formation of a $R_{3}^{2}\left(10\right)$ synthon, in this case through a strong OH···O H-bond and a weaker CH···S H-bond. Moreover, a $R_{2}^{2}\left(6\right)$ heterosynthon, where the OH acts as an H-bond donor and acceptor (–20.86 kJ/mol), is also generated. In addition, the ORC ring participates in a CH···π interaction involving the methyl group of PTU that is characterized by a large RDG isosurface that embraces the whole π-system, as is typical in this type of interaction. For RES (Figure 13), one binding mode implies the formation of a bifurcated OH···S,O, and an ancillary CH···O H-bonds with two different PTU molecules, generating $R_{2}^{2}\left(6\right)$ and $R_{2}^{2}\left(8\right)$ synthons. The other binding mode observed for RES generates a $R_{3}^{2}\left(10\right)$ synthon (similar to ORC) via the formation of a strong OH···O H-bond and a weaker CH···S H-bond, as well as an additional $R_{2}^{2}\left(6\right)$ heterosynthon, where the OH acts as both an H-bond donor and acceptor (–19.52 kJ/mol). In this cocrystal, the RES ring also participates in a CH···π interaction involving the methyl group of PTU, as revealed by the NCIplot analysis. The energy of the assemblies represented in Figure 12 and Figure 13 are similar in both cases (–145.0 kJ/mol and –135.8 kJ/mol for PTU–ORC and PTU–RES cocrystals, respectively), as expected, taking into consideration that both cocrystals present similar synthons in the solid state. 

**Figure 14:**
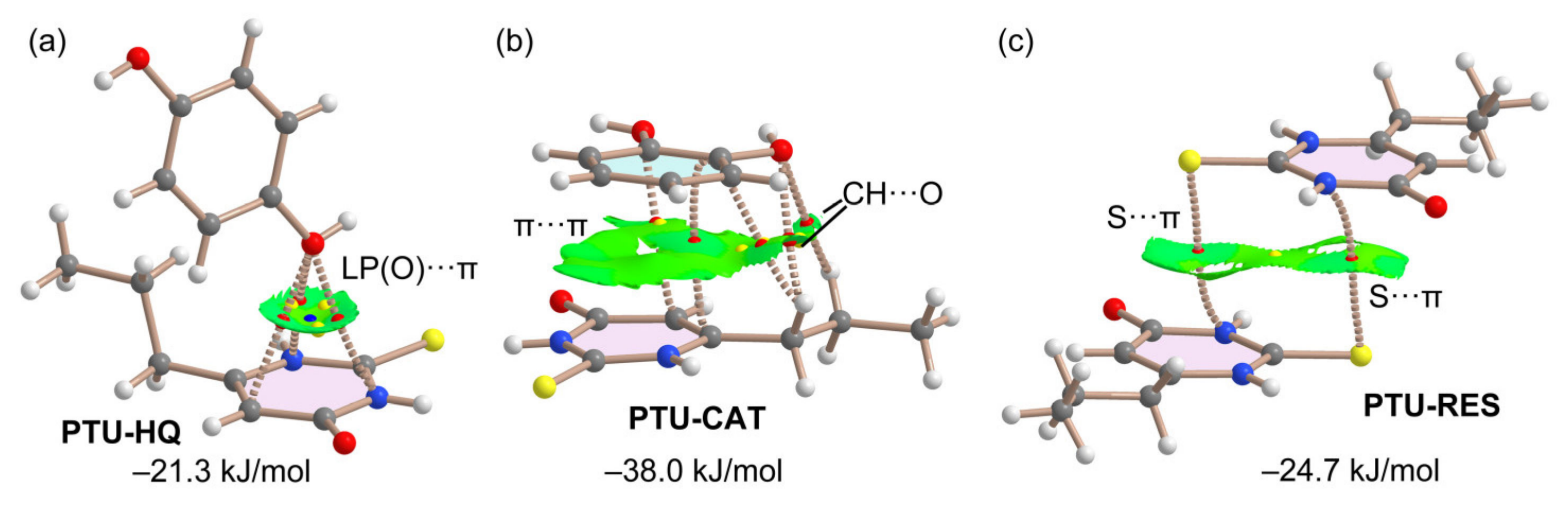
QTAIM/NCIPlot analysis of intermolecular bond, ring, and cage CPs (red, yellow and cage spheres, respectively), and bond paths and RDG isosurfaces of the π-assemblies of PTU–HQ (**a**), PTU–CAT (**b**), and PTU–RES (**c**) cocrystals.

Some of the cocrystals described above exhibit π-interactions that are relevant to understand their crystal packing. In particular, lone pair O···π, π-stacking and S···π interactions are observed in PTU–HQ, PTU–CAT, and PTU–RES, respectively, where the 2-thiouracil ring acts as an electron acceptor in agreement with the MEP analysis (Figure 9). The assemblies have been analyzed theoretically by computing the dimerization energies (supramolecular approach) and also using the QTAIM and NCIplot methods combined. The results are gathered in Figure 14, where the QTAIM analysis confirms the existence of the LP(O)···π interaction in PTU–HQ, which is characterized by three bond CPs and bond paths connecting the O-atom to three atoms of the 2-thiouracil ring. The interaction is further characterized by three ring CPs (yellow spheres) and one cage CP (blue sphere), as well as a green RDG isosurface that embraces the whole six-membered ring, as is typical in π-interactions. The π-stacked dimer in PTU–CAT is characterized by several bond CPs and bond paths interconnecting the coformers. The RDG isosurface embraces the whole region between the π-systems and extends toward the propyl substituent, revealing the existence of some extra contacts. Finally, in PTU–RES, the existence of the symmetrically equivalent S···π interactions is also confirmed by two bond CPs and bond paths interconnecting the S and N-atoms of 2-thiouracil rings. The S···π character of the interaction is better described by the NCIplot analysis since the RDG isosurface extends to the whole regions between the 6-membered ring and the sulfur atom. The dimerization energies are also indicated in Figure 14, showing that the LP···π interaction in PTU–HQ is –21.3 kJ/mol, similar to the double S···π interaction in PTU–RES (–24.7 kJ/mol). Such binding energies are smaller (in absolute value) than the association energies of the H-bonded synthons shown above. For the π-stacking in PTU–CAT, the binding is stronger (–38.0 kJ/mol) because of the large overlap of the π-systems, the electrostatic attraction between the electron-rich π-system of CAT (see Figure 9), and the electron-poor character of the 2-thiouracil ring.

### 2.6. FT-IR Spectroscopy

FT-IR spectroscopy offers information about the formation of non-covalent interactions between functional groups that build the cocrystal structure; therefore, it is a widely used technique to determine the formation of novel multicomponent materials [36]. The interactions between functional groups are reflected as shifts in the characteristic FT-IR band of the corresponding groups. In this work, a previous study of the crystalline structure through SCXRD has revealed the formation of intermolecular hydrogen bonds involving N–H, C=O and C=S groups of PTU and OH- groups of polyphenol molecules. Therefore, shifts in the bands ascribed to these functional groups are expected in the FT-IR analysis. Figure S4 shows the principal peaks of PTU and the respective shifts in the cocrystal spectrum, which are summarized in Table 2 for better understanding. These data indicate the formation of multicomponent materials, corroborating the powder and single-crystal X-ray diffraction results.

### 2.7. Thermal Analysis

**Figure 15:**
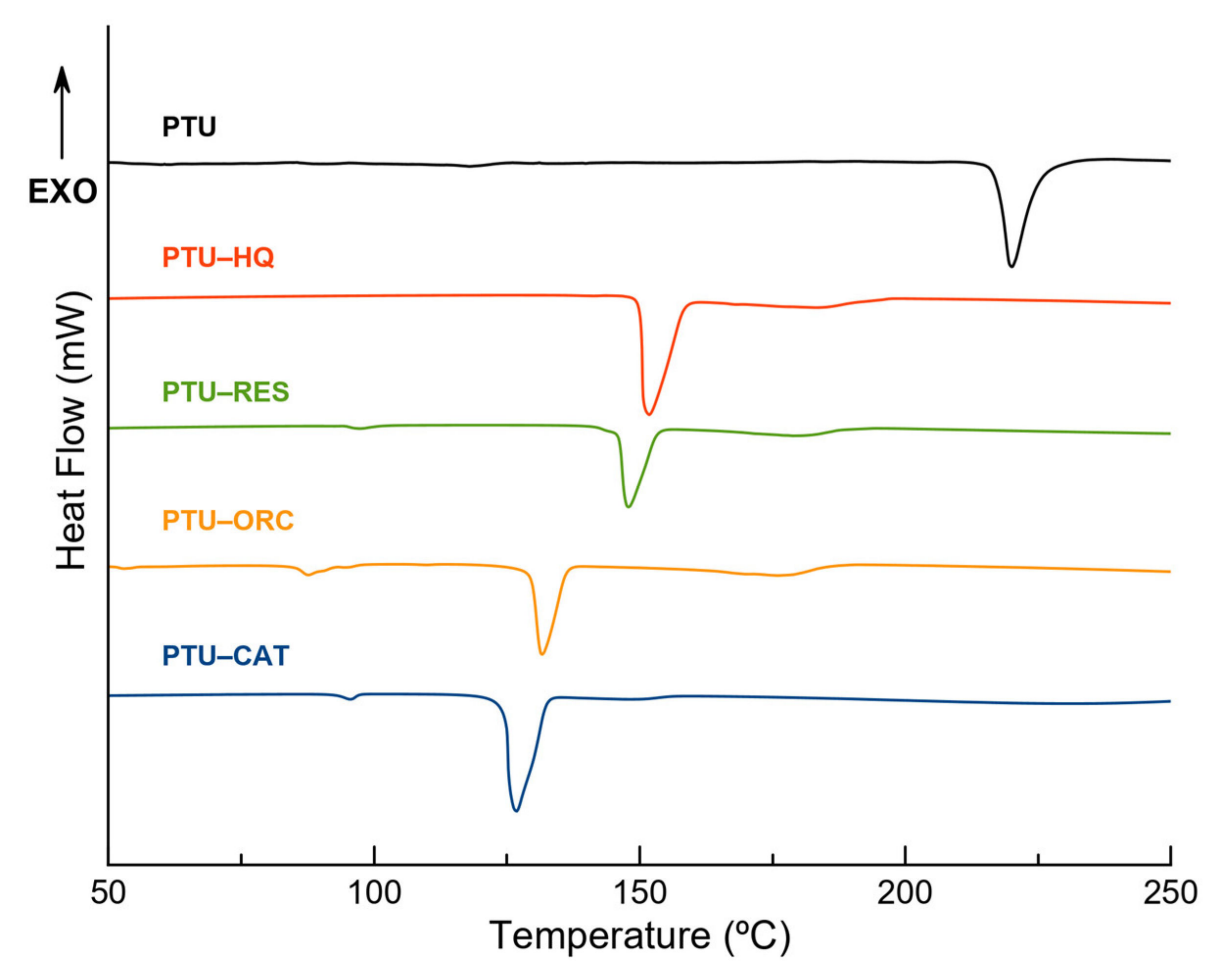
DSC traces of PTU and the new cocrystals.

Simultaneous differential scanning calorimetry (DSC) and thermogravimetric (TG) analysis were used to study the thermal behavior and the melting point of the reported cocrystals. Figure 15 shows the DSC traces of the cocrystals and the endothermic events occurring during the experiments. Low-energy endothermic events are observed within the range of 87–97 °C in PTU–RES, PTU–ORC, and PTU–CAT. These events can be ascribed to a phase transition since TG analysis does not show any mass loss related to these events (Figure S5), and the melting points of the respective coformers do not match the temperature region in question (CAT: 105 °C, ORC: 106–112 °C, RES: =110 °C, and HQ: =172 °C).

The most intense endothermic event in the experiments is attributed to the melting point of the new cocrystals, which falls in between the melting point of PTU (220 °C) and the corresponding coformer. A positive correlation between the melting point of the coformer and the cocrystal phase is also observed: as the melting point of the coformer molecule increases in the order of CAT < ORC < RES < HQ, the melting point of the cocrystals also increases in the order of PTU–CAT (126 °C) < PTU–ORC (132 °C) < PTU–RES (148 °C) < PTU–HQ (152 °C). This modulation of the thermal stability is in good agreement with the usual behavior of multicomponent materials, which has already been observed and reported in different studies [37].

After the melting point, TG shows a mass loss related to the degradation of the phases. No other endothermic events below the melting point are observed, indicating the purity of the cocrystals, which is in good agreement with the results obtained by PXRD.

### 2.8. Stability

The thermodynamic stability of PTU cocrystals was studied by conducting aqueous slurry (pH 6.8 buffer phosphate) experiments at 25 °C. After 24 h, the suspensions were filtered, air-dried at room temperature, and characterized by PXRD. Powder patterns revealed that all cocrystals dissociate into their parent components, and precipitation of PTU was observed after 24 h in all the cases, with the coformers remaining in solution (Figure S6). PTU cocrystals were also stored at accelerating aging conditions (40 °C, 75% RH). Under these conditions, it was observed that only PTU–HQ remained stable for two months; meanwhile, the other cocrystals exhibited associations (Figure S7). The stability of the cocrystals at accelerated test conditions is consistent with the thermodynamic stability observed in the slurry experiments.

### 2.9. Powder Dissolution Profile

**Figure 16:**
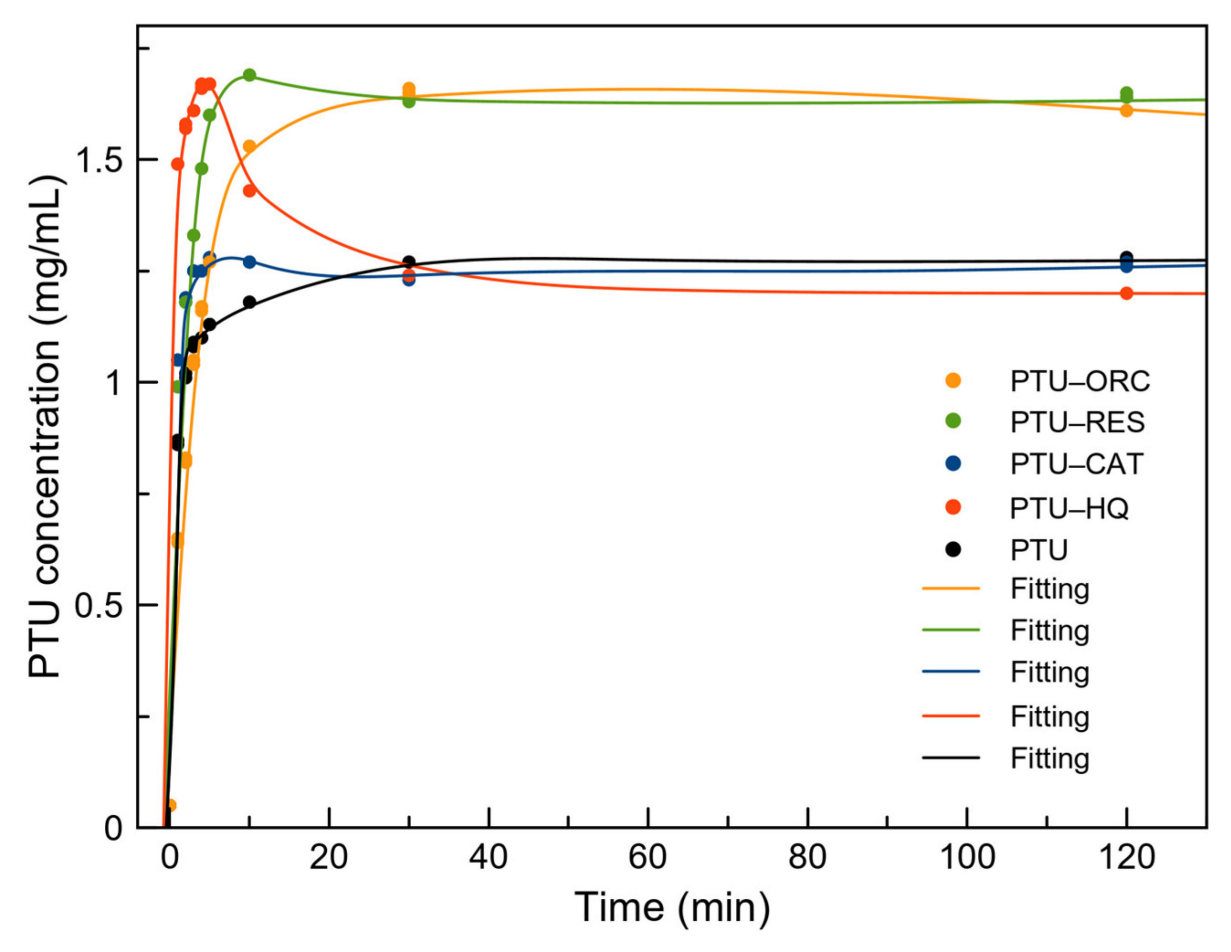
Powder dissolution profile of PTU and PTU cocrystals and the corresponding fitting curves (solid lines).

Powder dissolution experiments were conducted to evaluate the solubility and dissolution behavior of the PTU cocrystals in pH 6.8 phosphate buffer medium under a constant temperature and stirring rate. Powder dissolution profiles of PTU and the new PTU cocrystals are shown in Figure 16 as PTU concentrations (mg/mL) against time (min). The high stirring rate of 600 rpm applied to the crystals should result in complete dispersion, establishing a true equilibrium [38,39]. The maximum apparent solubilities (S_max_) of PTU and the new cocrystals are summarized in Table S4. As can be observed, the PTU cocrystals with RES and ORC can achieve a higher PTU concentration with a faster rate (around 1.3× at 30 min). PTU–HQ exhibits a “spring and parachute” behavior, reaching a high PTU concentration (1.48×) at 5 min and then decreasing its PTU concentration below the parent API concentration (0.98×). Meanwhile, PTU–CAT reaches a similar concentration as PTU. The supersaturated solutions were formed at the beginning of the dissolution process, and then the concentrations of the cocrystals kept almost constant up to the end of the experiment at 120 min. The PXRD analysis of the remaining powder after the dissolution experiments confirms the partial phase conversion of the cocrystals to pure PTU. Interestingly, the higher S_max_ combined with the absence of a “spring and parachute effect” in the dissolution profile of PTU–RES and PTU–ORC cocrystals presents an opportunity for using these crystal phases as potential immediate release forms [40], thereby reducing the amount of API in the formulation.

A plausible explanation of the observed solubility trend can be obtained from the crystal structural analysis on the basis of the role of the position of the hydroxy substituents of the polyphenolic coformers on the supramolecular arrangement. PTU–HQ cocrystals reach a higher PTU concentration at a faster rate while exhibiting longer chain separation. Conversely, PTU–RES and PTU–ORC cocrystals at the time show better apparent solubility than PTU–CAT cocrystals, with the latter displaying a higher PTU concentration at 5 min but then decreasing below the API itself. This can be attributed to the disruption of PTU chains in the crystal structure in favor of the formation of a robust tetrameric structure.

## 3. Materials and Methods

### 3.1. Materials

PTU was commercially available from TCI Europe (Zwijndrecht, Belgium), and all the coformers were commercially available from Sigma-Aldrich (purity > 98%, Sigma-Aldrich, St. Louis, MO, USA). All solvents were also purchased from Sigma-Aldrich and were used as received.

### 3.2. Cocrystal Synthesis

Mechanochemical synthesis was conducted by liquid-assisted grinding (LAG) in a Retsh MM2000 ball mill, operating at 25 Hz frequency, using stainless steel jars along with two stainless steel balls of 7 mm diameter. All reaction syntheses lasted for 30 min and were repeated to ensure reproducibility.

PTU–CAT was obtained by LAG of a mixture of PTU (0.5 mmol, 88.12 mg) and CAT (0.5 mmol, 55.05 mg) in a 1:1 stoichiometric ratio, using 150 µL of dichloromethane as a liquid additive. 

PTU–RES, PTU–HQ and PTU–ORC were obtained by LAG of a mixture of PTU (1 mmol, 170.25 mg) and the respective coformer (0.5 mmol, 55.05 mg of RES or HQ and 0.5 mmol, 62.06 mg of ORC) in a 2:1 stoichiometric ratio, using 150 µL of dichloromethane as a liquid additive.

After 30 min of milling, the bulk materials were collected and evaluated by PXRD to determine the formation of new materials.

Single crystals of the phases were obtained from a saturated solution of the LAG product in dichloromethane (for PTU–CAT and PTU–HQ) and ethanol (for PTU–RES and PTU–ORC). Suitable crystals for SCXRD appeared after 2 days of slow solvent evaporation at room temperature.

### 3.3. X-ray Diffraction Analysis

Measured crystals were prepared under inert conditions and immersed in perfluoropolyether as protecting oil for manipulation. Suitable crystals were mounted on MiTeGen Micromounts (MiTeGen, Ithaca, NY, USA), and these samples were used for data collection. Data for the reported cocrystals were collected with a Bruker D8 Venture diffractometer (Bruker-AXS, Karlsruhe, Germany) with graphite monochromated CuKα radiation (λ = 1.54178 Å) or Ag-Kα radiation (λ = 0.56086 Å) at 298(2) K. The data were processed with the APEX4 suite [41]. The structures were solved by intrinsic phasing using the ShelXT program [42], which revealed the position of all non-hydrogen atoms. These atoms were refined on F^2^ by a full-matrix least-squares procedure using anisotropic displacement parameters [43]. All hydrogen atoms were located in the difference Fourier maps and included as fixed contributions riding on attached atoms with isotropic thermal displacement parameters 1.2 or 1.5 times those of the respective atom. The Olex2 software (version 1.5) was used as a graphical interface [44]. Intermolecular interactions were calculated using PLATON [45]. Molecular graphics were generated using Mercury [35] and Olex2 [44]. The crystallographic data for the reported structures were deposited with the Cambridge Crystallographic Data Center as supplementary publication No. CCDC 2234883-2234886. Additional crystal data are shown in Table S1. Copies of the data can be obtained free of charge at https://www.ccdc.cam.ac.uk/structures/, accessed on 6 February 2023.

Powder X-ray diffraction (PXRD) data was collected on a Bruker D8 Advance Vαrio diffractometer (Bruker-AXS, Karlsruhe, Germany) diffractometer equipped with a LYNXEYE detector and CuKα_1_ radiation (1.5406 Å). The diffractograms were collected over an angular range of 5–40° (2θ) with a step size of 0.02° (2θ) and a constant counting time of 5 s per step.

### 3.4. Theoretical Methods

The assemblies shown in Figure 10, Figure 11, Figure 12, Figure 13 and Figure 14 were computed using Gaussian-16 [46,47] at the PBE0 [48]-D3 [48]/def2-TZVP [49] level of theory. To evaluate the different synthons and CH···O interactions, we used the quantum theory of “atoms-in-molecules” [50,51] at the same level by means of the AIMAll program [52]. To do so, we used the kinetic energy density values at the bond critical points that emerge upon complexation and applied the methodology proposed by Espinosa et al. [53]. This methodology has been recently used by some of us to evaluate non-covalent interactions in the solid state [54,55,56,57,58,59,60,61]. The NCIplot isosurfaces [62,63] have been generated using the AIMAll program [52] using the PBE0-D3/def2-TZVP wavefunction. The MEP surface pots have been performed using the 0.001 a.u. isosurface as the best estimate of the van der Waals envelope.

### 3.5. FT-IR Analysis

Fourier-transform infrared (FT-IR) spectroscopy measurements of the cocrystals were performed on a Bruker Tensor 27 FT-IR instrument (Bruker Corporation, Billerica, MA, USA) equipped with a single-reflection diamond crystal platinum ATR unit and OPUS data collection program. The scanning range was from 4000 to 400 cm^–1^ with a resolution of 4 cm^–1^.

### 3.6. Thermal Analysis

Simultaneous differential scanning calorimetry (DSC) and thermogravimetric analysis were performed using a Mettler Toledo TGA/DSC1 thermal analyzer (Mettler Toledo, Columbus, OH, USA). Samples (3–5 mg) were placed into sealed aluminum pans and heated in a stream of nitrogen (100 mL min^–1^) from 25 to 400 °C at a heating rate of 10 °C/min. The calorimeter was calibrated with indium of 99.99% purity (m.p.: 156.4 °C; DH: 28.14 J/g).

### 3.7. Stability Studies

The thermodynamic stability of the PTU cocrystals in aqueous solution was evaluated through slurry experiments. An excess of powder samples of each phase was added to 1 mL of buffer phosphate (pH 6.8) and stirred for 24 h in sealed vials. The solids were collected, filtered, dried at 35 °C and further analyzed by PXRD. The cocrystals were also stored under accelerated aging conditions (40 °C and 75% RH), placing powder samples in a Memmert HPP110 climate chamber (Memmert, Schwabach, Germany) for up to 2 months. The samples were analyzed by PXRD periodically to evaluate possible phase transformations during the time of the experiment.

### 3.8. Powder Dissolution Profile

Samples for the powder dissolution profile studies were prepared following the shake-flask method [64]. Saturated solutions of each solid were prepared by adding a solid excess to 15 mL of PBS buffer solution at pH 6.8. This pH value corresponds to that observed in the small intestine, where the vast majority of the drug is meant to be absorbed. The solution was stirred for 24 h at 25 °C in a water bath until thermodynamic equilibrium was reached. During this time, aliquots of 1 mL were taken at different times, filtered through 0.22 μm polyether sulfone (PES) filters, and directly measured by high-performance liquid chromatography (HPLC). The HPLC calibration curve of PTU is given in Figure S8. Appropriate dilutions were made to obtain measurable absorbance values. The absorbance measurements were thereafter used to quantify the PTU solubilized in each sample. The remaining solids were analyzed by PXRD to identify the crystalline phases and, thus, to check the stability of the initial crystalline phase.

HPLC experiments were performed with an Agilent 1260 Infiniti II HPLC system (Agilent Technologies, Santa Clara, CA, USA) using a Waters Atlantis T3 chromatographic column (5 μm, 100 × 4.6 mm) at 40 °C. The mobile phase was composed of two solvents; solvent A was a mixture of 90% acetonitrile and 10% water (13 mM of ammonium formate and 0.01% of TFA *v*/*v*), and solvent B was a mixture of 10% acetonitrile and 90% water (13 mM of ammonium formate and 0.01% of TFA *v*/*v*). The flow rate was 0.5 mL/min, and the injection volume was 2μL. The absorbance was measured at 276 nm (maximum absorbance for PTU). Data acquisition and analysis were performed using the software ChemStation (Agilent Technologies, Santa Clara, CA, USA). The retention time for PTU was 4 min 58 s, and the concentration for the calibration curve was determined from the area under the PTU peak.

## 4. Conclusions

DFT and SCXRD analysis revealed the formation of two types of recurrent $R_{2}^{2}\left(8\right)$ motifs. They consist of PTU homodimers with two weaker NH···S H-bonds (ranging from 4.93 to 5.30 kJ/mol) and two stronger NH···O bonds (ranging from 10.73 to 12.30 kJ/mol). The formation of the latter is also well supported by the MEP analysis since PTU presents the better H-bond donor and acceptor groups. In the crystal structure of PTU–HQ, PTU–RES, and PTU–ORC cocrystals, the referred N-H···S homosynthons generate infinite chains similar to that described in the crystal structure of PTU alone. Nevertheless, in the novel multicomponent solids, PTU chains are further connected by the corresponding coformers, with the distance between chains varying according to the relative position of the -OH groups in the different polyphenol isomers (HQ > RES = ORC). Note that cocrystals with RES and ORC coformers are iso-structural; hence, they exhibit similar supramolecular architectures. On the other hand, PTU–CAT shows a different structural arrangement in which two CAT coformers build a tetrameric structure with PTU, thus preventing the formation of the PTU chains described above. This fact is certainly related to the -ortho position of the -OH substituents in CAT. The QTAIM/NCIplot analysis also reveals the existence of $R_{2}^{1}\left(6\right)$ and $R_{2}^{2}\left(6\right)$ synthons involving the phenol groups that are relevant energetically (up to 9.63 kJ/mol in PTU–CAT). In addition, some interesting π-interactions have also been described, where the π-system of PTU acts as an electron acceptor. The interaction energies are moderately strong, ranging from –5.1 kJ/mol for the LP···π interaction in PTU–HQ to –9.1 kJ/mol for the electrostatically enhanced π-stacking interaction in PTU–CAT. 

Regarding their physicochemical properties, all novel cocrystals except PTU–CAT show solubility enhancement compared to PTU alone. Nevertheless, greater values are observed for PTU–RES and PTU–ORC cocrystals, which is consistent with the association energy values calculated for their global assemblies (PTU–RES 32.5 KJ/mol and PTU–ORC 34.7 KJ/mol vs. PTU–CAT 42.5 KJ/mol and PTU–HQ 50.7 KJ/mol). This is consistent with the analysis of the crystal structure, where PTU chains formed in PTU–RES and PTU–ORC show the shortest distance. Moreover, PTU–RES and PTU–ORC show interesting immediate release profiles, reaching their maximum concentration within 30 min. Although the thermal stability of the novel phases is rather good, their thermodynamic stability in aqueous media leaves room for improvement, demonstrating the association of the multicomponent solids after 24 h. Unfortunately, their stability under aging conditions is also insufficient and needs to be improved to reach more suitable storage conditions for industry. 

## Figures and Tables

**Table 1:** Ranking positions for coformers reported at CSD that form multicomponent solids with PTU, including the coformers used in this study (in bold) based on COSMOQuick calculations.

Coformer	H_ex_ [kJ/mol]	Reference
**Catechol**	**−2.081625**	**This work**
**Resorcinol**	**−2.04305**	**This work**
Kaempferol	−1.910405	[25]
Gentisic acid	−1.78116	[25]
**Orcinol**	**−1.77036**	**This work**
Ellagic acid	−1.717475	[25]
**Hydroquinone**	**−1.477705**	**This work**
2,4-Diaminopyrimidine	−0.294005	[29]
N-(6-acetamidopyridin-2-yl)acetamide	−0.25925	[28]
2-Aminopyrimidine	−0.20317	[28]
Sinapic acid	−0.17387	[27]
Cinnamic acid	−0.14058	[25]

**Table 2:** Characteristic FT-IR bands of PTU and their attribution in PTU alone and the obtained cocrystals.

	Wavenumber (cm^–1^)		
Compound	C=O Stretching	N-H Bending	C=S Stretching
PTU	1651.94	1557.44	1190.98
PTU–CAT	1660.61	1557.44	1184.23
PTU–RES	1646.16	1558.40	1180.37
PTU–HQ	1651.95	1563.22	1189.05
PTU–ORC	1651.94	1547.43	1201.58

## Data Availability

The Crystallographic Information File with the structural data of the new phase can be obtained from the CCDC and requested with the references 2234883-2234886 at https://www.ccdc.cam.ac.uk/structures/, accessed on 6 February 2023.

## Supplementary Materials

The following supporting information can be downloaded at: https://www.mdpi.com/article/10.3390/ph16030370/s1, Figure S1: PXRD patterns of the PTU cocrystals obtained by LAG, compared with their respective components; Figure S2: PXRD patterns of PTU cocrystals obtained by grinding the two components at different molar ratios; Figure S3: ORTEP representation showing the asymmetric unit of PTU–CAT (a), PTU–RES (b), PTU–ORC (c) and PTU–HQ with atom numbering scheme (thermal ellipsoids are plotted with the 50% probability level); Figure S4. FT-IR spectrum of pure PTU and the cocrystals reported; Figure S5. DSC-TG traces of (a) ETZ–CAT, (b) ETZ–RES, (c) ETZ–ORC and (d) ETZ–HQ; Figure S6: PXRD patterns of the reported PTU cocrystals after aqueous slurrying (pH 6.8 phosphate buffer medium) for 24 h; Figure S7: PXRD patterns of reported molecular salts under accelerated aging conditions for 2 months; Figure S8: Calibration curve of PTU determined from HPLC data; Table S1: Crystallographic data and structure refinement details of PTU cocrystals; Table S2: Hydrogen bonds for PTU cocrystals (Å and deg.); Table S3: π-stacking interactions analysis of PTU cocrystals; Table S4: Maximum apparent solubility (S_max_) of PTU and its cocrystals in pH 6.8 phosphate buffer medium.
