# Supplementary material for: Rational Coformer Selection in the Development of 6-Propyl-2-thiouracil Pharmaceutical Cocrystals
Source: Pharmaceuticals (Basel). 2023 Feb 28;16(3):370. doi: 10.3390/ph16030370 (PMC10055029; doi:10.3390/ph16030370)
Supplement: Supplementary file 1 [file pharmaceuticals-16-00370-s001.zip › pharmaceuticals-2237682-supplementary.pdf]

Table of contents

Figure S1. PXRD patterns of the PTU cocrystals obtained by LAG, compared with their respective components.
Figure S2. PXRD patterns of PTU cocrystals obtained by grinding the two components at different molar ratios.
Figure S3. ORTEP representation showing the asymmetric unit of PTU—CAT (a), PTU—RES (b), PTU—ORC (c) and PTU—HQ with atom numbering scheme (thermal ellipsoids are plotted with the 50% probability level).
Figure S4. FT-IR spectrum of pure PTU and the cocrystals reported.
Figure S5. DSC-TG traces of a) ETZ–CAT, b) ETZ–RES, c) ETZ–ORC and d) ETZ–HQ.
Figure S6. PXRD patterns of the reported PTU cocrystals after aqueous slurrying (pH 6.8 phosphate buffer medium) for 24 hours.
Figure S7. PXRD patterns of reported molecular salts under accelerated ageing conditions for 2 months.
Figure S8. Calibration curve of PTU determined from HPLC data.

Table S1. Crystallographic data and structure refinement details of PTU cocrystals.
Table S2. Hydrogen bonds for PTU cocrystals [Å and deg.].
Table S3. π-stacking interactions analysis of PTU cocrystals.
Table S4. Maximum apparent solubility (S_{max}) of PTU and its cocrystals in pH 6.8 phosphate buffer medium.

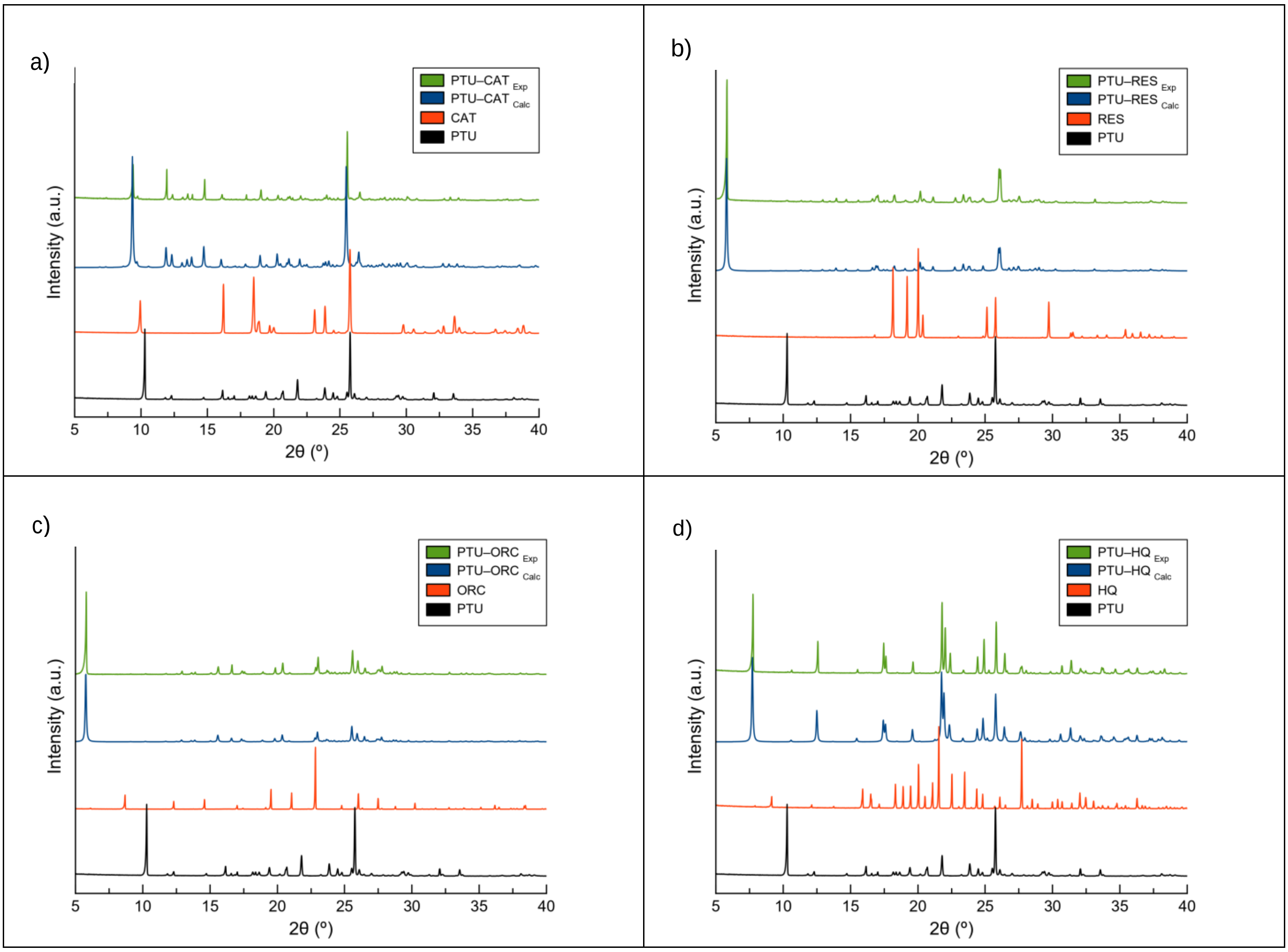

Figure S1. PXRD patterns of the PTU cocrystals obtained by LAG, compared with their respective components.

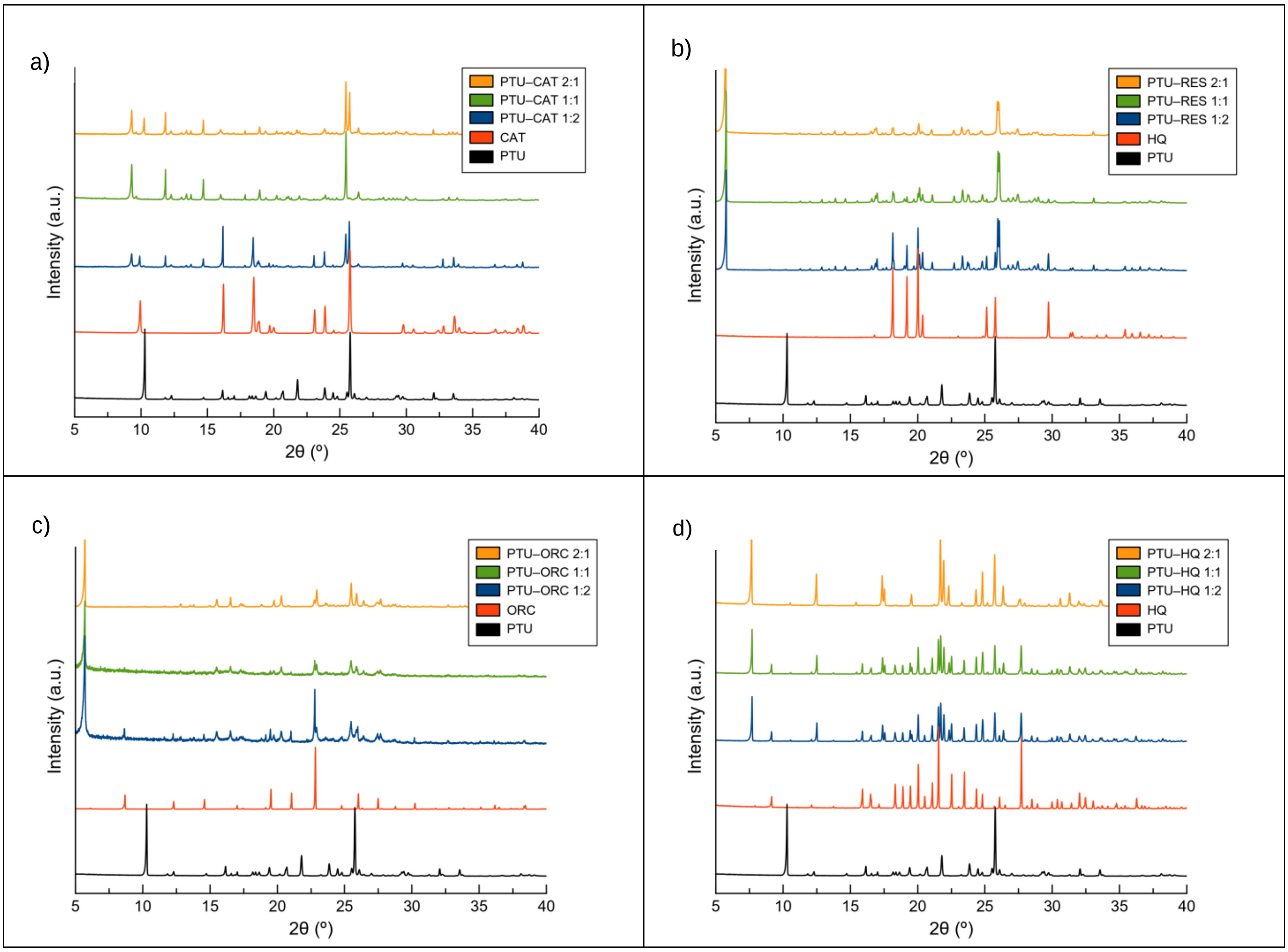

Figure S2. PXRD patterns of PTU cocrystals obtained by grinding the two components at different molar ratios.

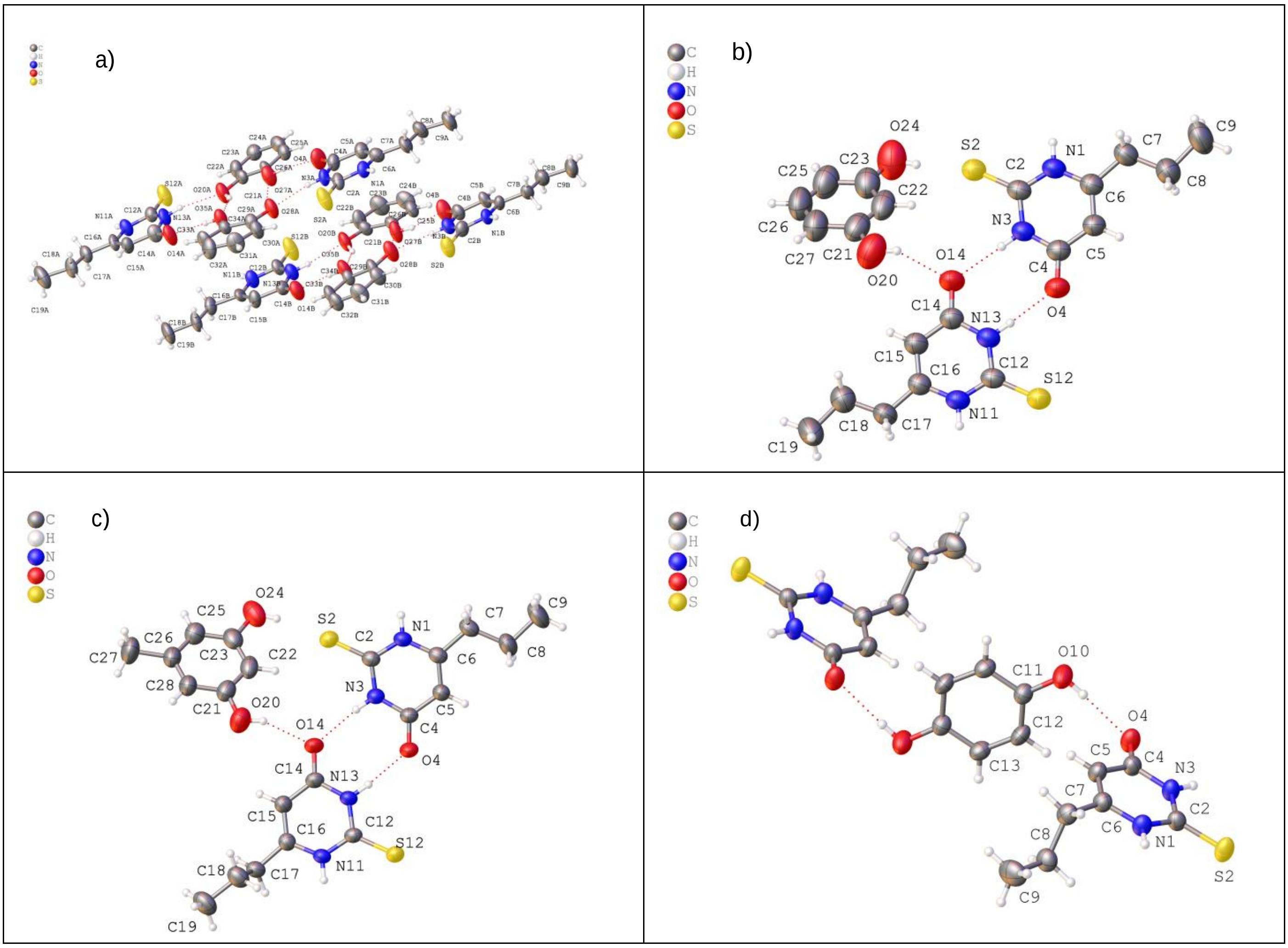

Figure S3. ORTEP representation showing the asymmetric unit of PTU—CAT (a), PTU—RES (b), PTU—ORC (c) and PTU—HQ with atom numbering scheme (thermal ellipsoids are plotted with the 50% probability level).

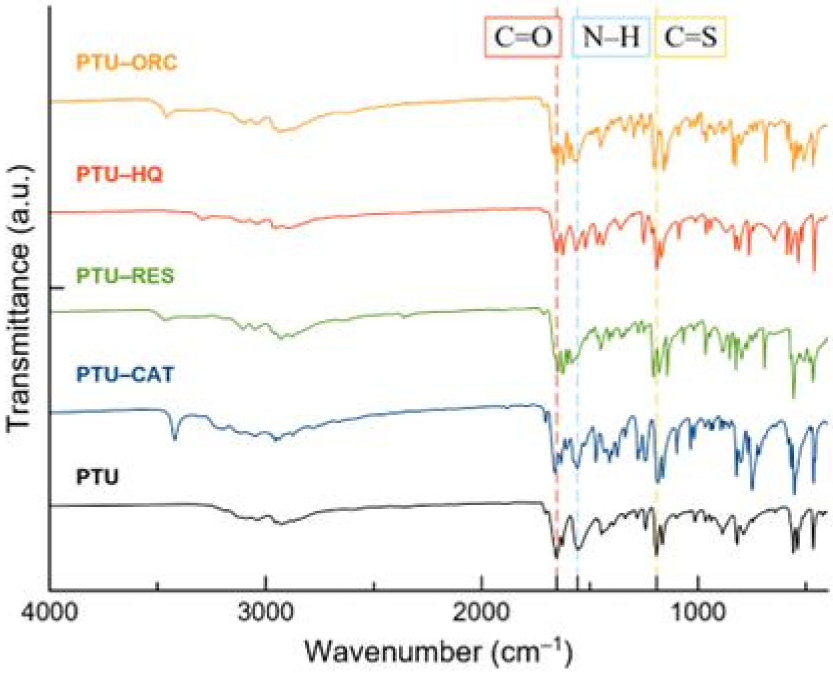

Figure 4. FT-IR spectrum of pure PTU and the cocrystals reported.

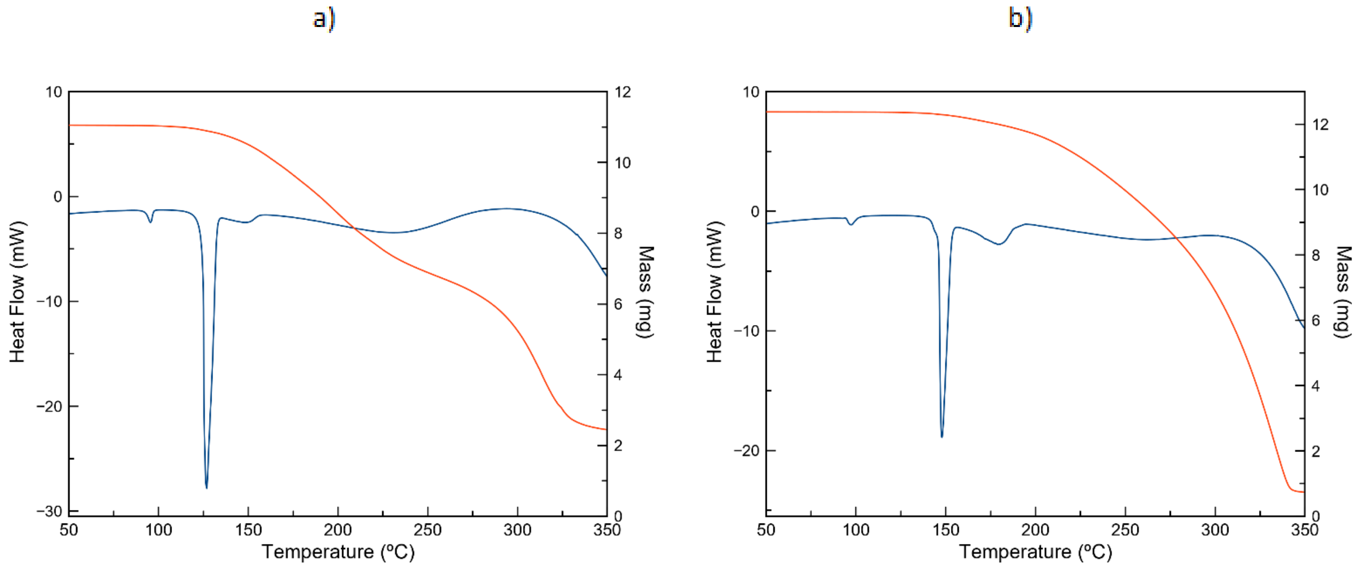

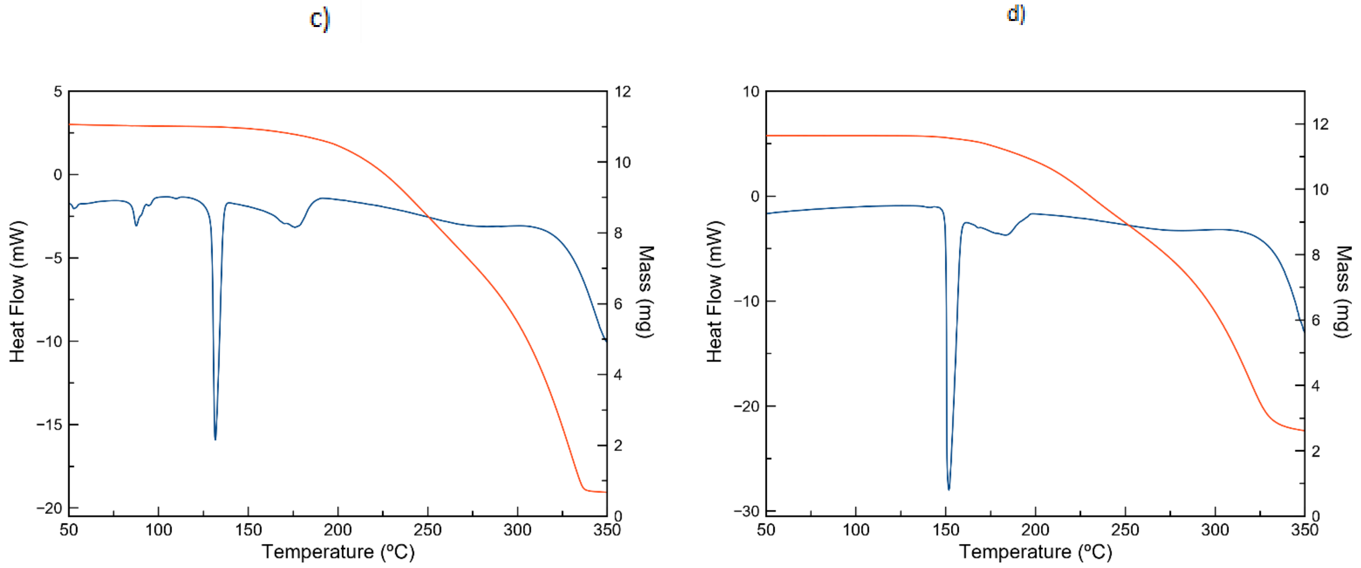

Figure S5. DSC-TG traces of a) ETZ–CAT, b) ETZ–RES, c) ETZ–ORC and d) ETZ–HQ.

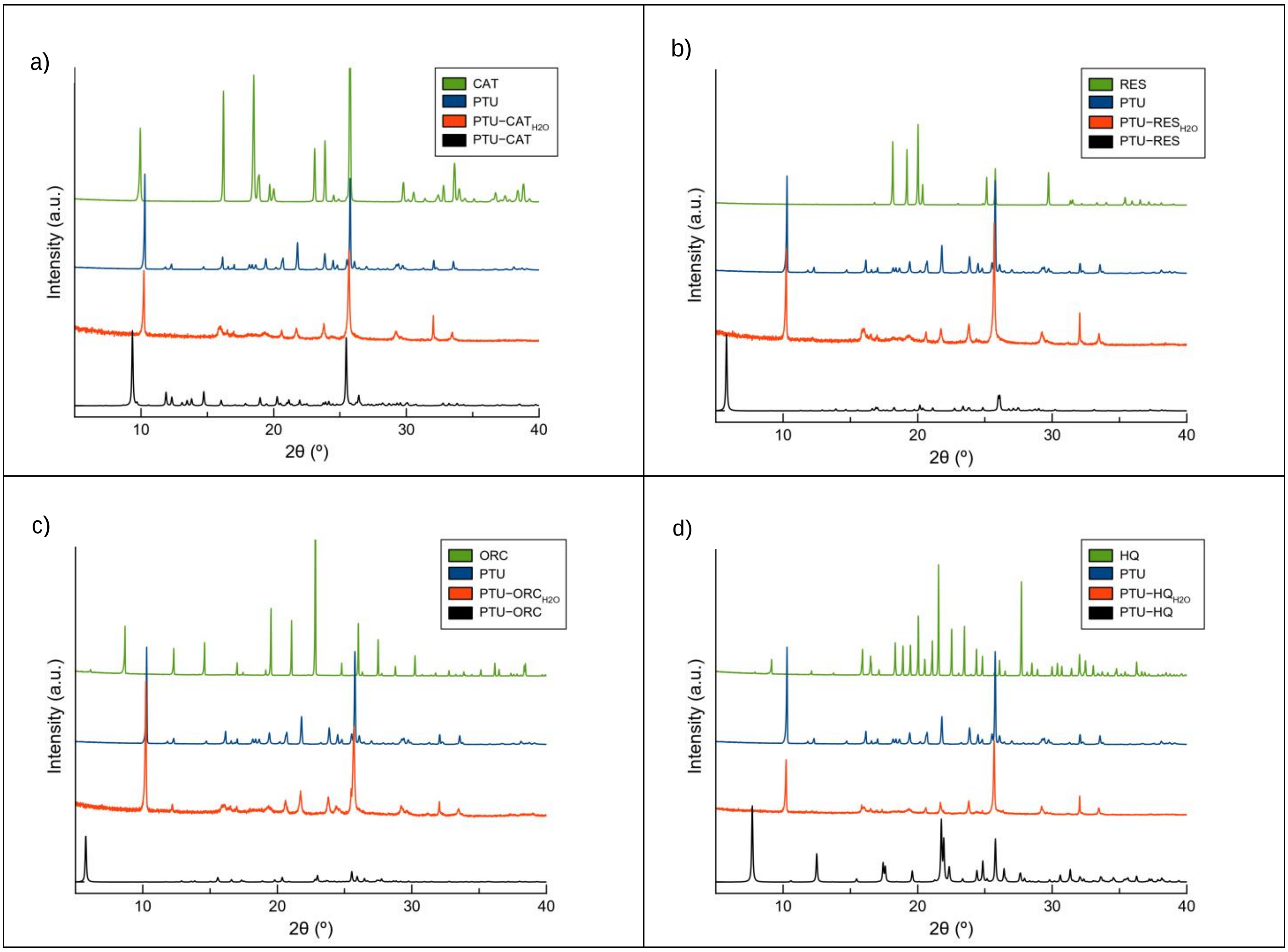

Figure S6. PXRD patterns of the reported PTU cocrystals after aqueous slurrying (pH 6.8 phosphate buffer medium) for 24 hours.

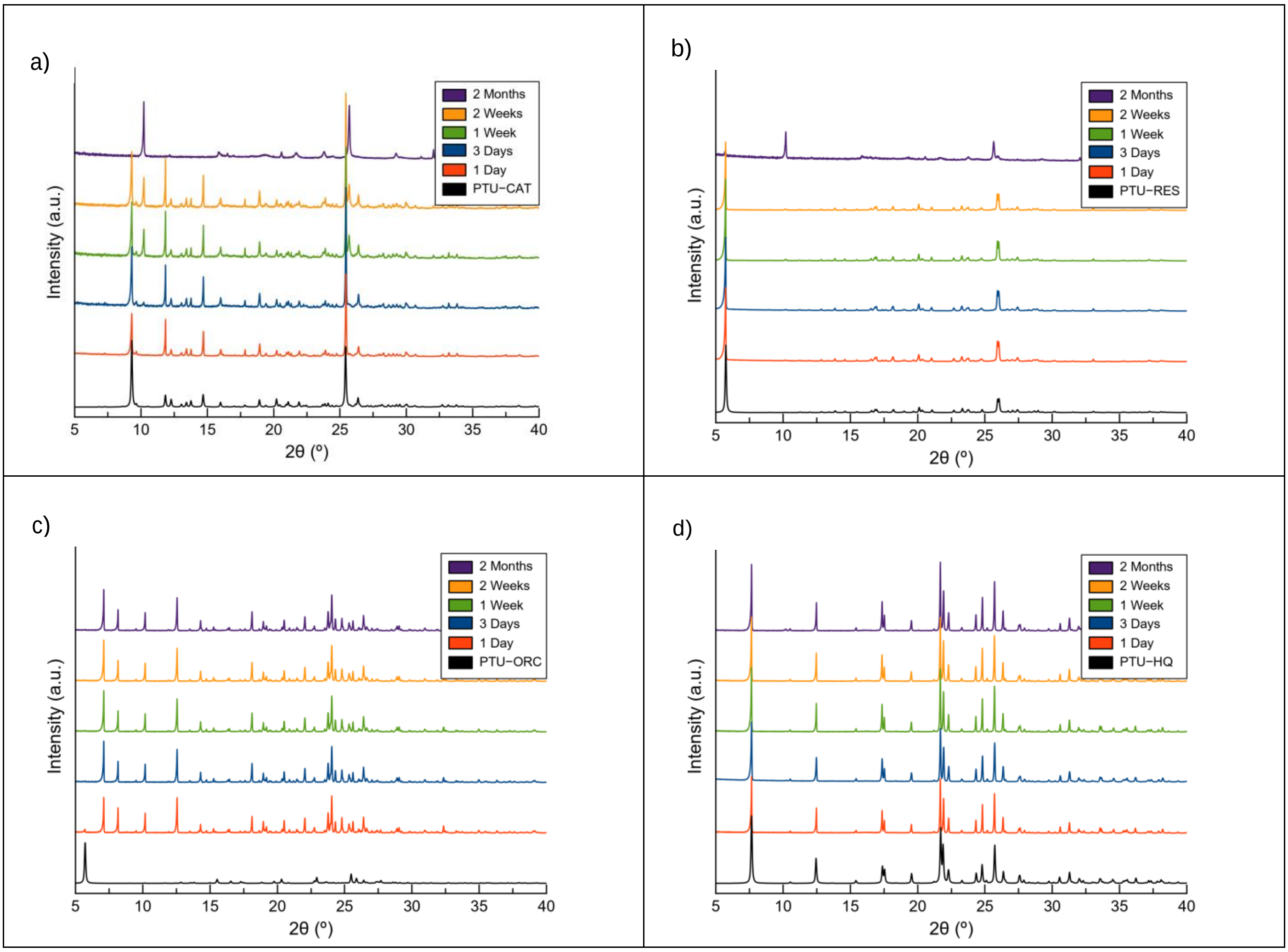

Figure S7. PXRD patterns of reported molecular salts under accelerated ageing conditions for 2 months.

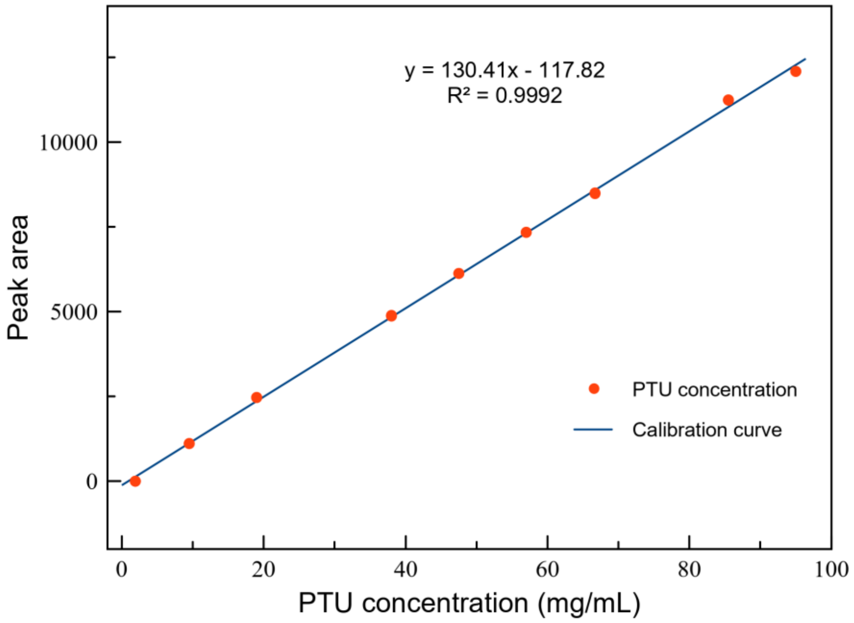

Figure S8. Calibration curve of PTU determined from HPLC data.

Table S1. Crystallographic data and structure refinement details of PTU cocrystals.

Identification code	PTU—CAT	PTU—RES	PTU—HQ	PTU—ORC
Formula	$C_{13}H_{16}N_2O_3S$	$C_{20}H_{26}N_4O_4S_2$	$C_{10}H_{13}N_2O_2S$	$C_{21}H_{28}N_4O_4S_2$
Formula weight	280.34	450.57	450.57	464.59
Crystal system	Monoclinic	Triclinic	Triclinic	Triclinic
Space group	$P2_1$	P-1	P-1	P-1
a/Å	13.701(5)	7.6530(3)	5.777(3)	7.8859(6)
b/Å	14.002(5)	10.2143(4)	8.491(3)	10.1513(7)
c/Å	15.910(6)	15.3443(7)	11.517(5)	15.5615(13)
α/°	90	82.998(2)	84.18(2)	81.425(3)
β/°	110.549(6)	87.179(3)	87.16(2)	81.944(4)
γ/°	90	73.614(2)	81.02(2)	74.319(3)
V/Å^3	2858.0(17)	1142.03(8)	554.8(4)	1179.42(16)
Z	8	2	1	2
D_c/g cm^{-3}	1.303	1.310	1.348	1.308
F(000)	1184	476	238	492
Reflections collected	38488	13620	6215	19123
Unique reflections	9446	3978	1886	4133
Data / restraints / parameters	9446 / 1 / 693	3978 / 0 / 275	1886 / 0 / 139	4133 / 0 / 285
Goodness-of-fit on F^2	0.974	1.050	1.102	0.999
$R1$ (I > 2σ(I))	0.0682	0.0473	0.0325	0.0506
w$R2$ (I > 2σ(I))	0.1435	0.1264	0.0809	0.1097

Table S2. Hydrogen bonds for PTU cocrystals [Å and deg.].

	D-H···A	d(D-H)	d(H···A)	d(D···A)	<(DHA)
PTU—CAT	D-H···A	d(D-H)	d(H···A)	d(D···A)	<(DHA)
	N(3A)-H(3A)···O(28A)	0.86	2.15	2.989(11)	166.7
	N(3B)-H(3B)···O(28B)	0.86	2.18	3.040(11)	173.0
	N(13A)-H(13A)···O(20A)	0.86	2.20	3.046(11)	168.6
	N(13B)-H(13B)···O(20B)	0.86	2.14	2.994(12)	171.3
	O(20A)-H(20A)···O(35A)	0.82	2.04	2.753(10)	144.9
	O(27A)-H(27A)···O(4A)	0.82	1.83	2.643(12)	168.7
	O(20B)-H(20B)···O(35B)	0.82	2.07	2.768(11)	142.8
	O(27B)-H(27B)···O(4B)	0.82	1.87	2.679(11)	166.5
	O(28A)-H(28A)···O(27A)	0.82	2.10	2.774(10)	139.9
	O(35A)-H(35A)···O(14A)	0.82	1.86	2.677(11)	178.1
	O(28B)-H(28B)···O(27B)	0.82	2.05	2.719(10)	138.8
	O(35B)-H(35B)···O(14B)	0.82	1.84	2.657(11)	173.0
PTU—RES	D-H···A	d(D-H)	d(H···A)	d(D···A)	<(DHA)
	N(3)-H(3)···O(14)	0.86	1.98	2.837(3)	176.7
	N(13)-H(13)···O(4)	0.86	1.95	2.796(3)	166.9
	C(22)-H(22)···O(4)#1	0.93	2.59	3.197(4)	123.6
	O(20)-H(20)···O(14)	0.82	2.09	2.820(3)	148.4
	Symmetry transformations used to generate equivalent atoms: #1 -x+1,-y+1,-z+1				
PTU—HQ	D-H···A	d(D-H)	d(H···A)	d(D···A)	<(DHA)
	N(3)-H(3)···O(4)#1	0.86	1.98	2.839(2)	172.0
	O(10)-H(10)···O(4)	0.82	1.98	2.786(2)	168.2
	Symmetry transformations used to generate equivalent atoms: #1 -x+1,-y,-z+1				
PTU—ORC	D-H···A	d(D-H)	d(H···A)	d(D···A)	<(DHA)
	N(3)-H(3)···O(14)	0.86	1.99	2.846(4)	170.3
	N(13)-H(13)···O(4)	0.86	1.91	2.766(4)	174.4
	O(20)-H(20)···O(14)	0.82	2.07	2.809(4)	150.3

Table S3. π-stacking interactions analysis of PTU cocrystals.

PTU-CAT

6-Membered Ring (1)	N1	-->	C2	-->	N3	-->	C4	-->	C5	-->	C6	-->
6-Membered Ring (2)	N11	-->	C12	-->	N13	-->	C14	-->	C15	-->	C16	-->
6-Membered Ring (3)	C21	-->	C22	-->	C23	-->	C24	-->	C25	-->	C26	-->
6-Membered Ring (4)	C29	-->	C30	-->	C31	-->	C32	-->	C33	-->	C34	-->

Analysis of Short Ring-Interactions with Cg-Cg Distances < 6.0 Ang., Alpha < 20.000 Deg. and Beta < 60.0 Deg.

Cg(I)	Res(I)	Cg(J)	[ARU(J)]	Cg-Cg	Transformed J-Plane P, Q, R, S	Alpha	Beta	Gamma	CgI_Perp	CgJ_Perp	Slippage
Cg1	[1] ->	Cg1	[2565.01]	5.7733(18)	-0.9569 0.1140 0.2669 -0.5088	0.00(16)	56.6	56.6	3.1793(14)	3.1794(14)	4.819
**Cg1**	**[1] ->**	**Cg3**	**[1455.03]**	**3.748(2)**	**0.9289-0.2188-0.2987 -6.7221**	**6.48(17)**	**18.6**	**21.2**	**3.4932(13)**	**3.5520(15)**	**1.195**
Cg1	[1] ->	Cg3	[1555.03]	4.541(2)	0.9289-0.2188-0.2987 0.0810	6.48(17)	40.4	43.2	3.3099(13)	3.4564(15)	2.946
Cg2	[2] ->	Cg4	[1455.04]	4.509(2)	0.9582 0.0823-0.2742 -4.9344	5.36(16)	40.8	41.3	3.3867(13)	3.4123(15)	2.947
**Cg2**	**[2] ->**	**Cg4**	**[1555.04]**	**3.904(2)**	**0.9582 0.0823-0.2742 2.0829**	**5.36(16)**	**25.8**	**21.6**	**3.6305(13)**	**3.5142(15)**	**1.701**
Cg3	[3] ->	Cg1	[1555.01]	4.541(2)	0.9569-0.1140-0.2669 -2.6706	6.48(17)	43.2	40.4	3.4564(15)	3.3099(13)	3.109
**Cg3**	**[3] ->**	**Cg1**	**[1655.01]**	**3.748(2)**	**0.9569-0.1140-0.2669 4.3378**	**6.48(17)**	**21.2**	**18.6**	**3.5520(15)**	**3.4932(13)**	**1.357**
**Cg4**	**[4] ->**	**Cg2**	**[1555.02]**	**3.904(2)**	**0.9458 0.1749-0.2738 -0.3258**	**5.36(16)**	**21.6**	**25.8**	**3.5142(15)**	**3.6305(13)**	**1.436**
Cg4	[4] ->	Cg2	[1655.02]	4.509(2)	0.9458 0.1749-0.2738 6.6006	5.36(16)	41.3	40.8	3.4122(15)	3.3868(13)	2.977
			Min or Max	3.748		0.0	18.6	56.6	-3.631	-3.631	

[2565] = -X,1-Y,-Z [1455] = -1+X,Y,Z [1555] = X,Y,Z [1455] = -1+X,Y,Z [1555] = X,Y,Z [1555] = X,Y,Z [1655] = 1+X,Y,Z
[1555] = X,Y,Z [1655] = 1+X,Y,Z

Analysis of X-H...Cg(Pi-Ring) Interactions (H..Cg < 3.0 Ang. - Gamma < 40.0 Deg)

X--H(I)	Res(I)	Cg(J)	[ARU(J)]	H..Cg	Transformed J-Plane P, Q, R, S	H-Perp	Gamma	X-H..Cg	X..Cg	X-H,Pi
C7 -H7A	[1] ->	Cg3	[1555.03]	2.70	0.9289-0.2188-0.2987 0.0810	-2.70	2.38	143	3.525(3)	53
C17 -H17B	[2] ->	Cg4	[1455.04]	2.70	0.9582 0.0823-0.2742 -4.9344	2.69	4.75	140	3.501(4)	54
			Min or Max	2.700		-2.700	2.4	143.00	3.501	54.00

[1555] = X,Y,Z
[1455] = -1+X,Y,Z

Table S3 (cont.). π-stacking interactions analysis of PTU cocrystals.

PTU-RES

```
  6-Membered Ring ( 1)   N1     -->   C2     -->   N3     -->   C4     -->   C5     -->   C6     -->
  6-Membered Ring ( 2)   N11    -->   C12    -->   N13    -->   C14    -->   C15    -->   C16    -->
  6-Membered Ring ( 3)   C21    -->   C22    -->   C23    -->   C25    -->   C26    -->   C27    -->
=====================================================================================================

Analysis of X-H...Cg(Pi-Ring) Interactions (H..Cg < 3.0 Ang. - Gamma <  40.0 Deg)
=====================================================================================================

   X--H(I)    Res(I)   Cg(J)  [   ARU(J)]      H..Cg  Transformed J-Plane P, Q, R, S  H-Perp Gamma     X-H..Cg       X..Cg X-H,Pi

C19    -H19C   [ 2] -> Cg3    [  1645.03]       2.85   0.9487-0.3144 0.0344  10.1737  -2.79  11.35         170    3.800(4)    81
                                      ----------                         ---------------------------------------------
                              Min or Max  2.850                           -2.794   11.4      170.00       3.800  81.00
[  1645] = 1+X,-1+Y,Z

=====================================================================================================

Analysis of Y-X...Cg(Pi-Ring) Interactions (X..Cg < 4.0 Ang. - Gamma <  30.0 Deg)
=====================================================================================================

   Y--X(I)    Res(I)   Cg(J)  [   ARU(J)]      X..Cg  Transformed J-Plane P, Q, R, S  X-Perp Gamma     Y-X..Cg       Y..Cg Y-X,Pi

C2     -S2    [ 1] -> Cg1    [  2566.01]  3.4926(11)  -0.9024-0.4220-0.0873  -3.0087 -3.424  11.35    89.53(8)    3.855(3)   1.01
C12    -S12   [ 2] -> Cg2    [  2656.02]  3.6052(13)  -0.9225-0.3859 0.0059  -2.4193 -3.503  13.64    85.84(9)    3.861(3)   0.16
C14    -O14   [ 2] -> Cg1    [  2666.01]    3.422(2)  -0.9024-0.4220-0.0873  -9.9147  3.405   5.70   87.89(18)    3.600(3)   1.72
                                      ----------                         ---------------------------------------------
                              Min or Max  3.422                           -3.503    5.7       89.53       3.600   1.72

[  2566] = -X,1-Y,1-Z
[  2656] = 1-X,-Y,1-Z
[  2666] = 1-X,1-Y,1-Z
```

Table S3 (cont.). π-stacking interactions analysis of PTU cocrystals.

PTU-ORC

```
6-Membered Ring ( 1)  N1    -->  C2    -->  N3    -->  C4    -->  C5    -->  C6    -->
6-Membered Ring ( 2)  N11   -->  C12   -->  N13   -->  C14   -->  C15   -->  C16   -->
6-Membered Ring ( 3)  C21   -->  C22   -->  C23   -->  C25   -->  C26   -->  C28   -->
=====================================================================================================

Analysis of X-H...Cg(Pi-Ring) Interactions (H..Cg < 3.0 Ang. - Gamma <  40.0 Deg)
=====================================================================================================

  X--H(I)   Res(I)  Cg(J)  [   ARU(J)]     H..Cg  Transformed J-Plane P, Q, R, S  H-Perp Gamma    X-H..Cg      X..Cg X-H,Pi

C19   -H19C  [ 2] -> Cg3   [  1645.03]      2.90   0.9727-0.1556-0.1723 10.1114  -2.89   4.66       138    3.678(6)    53
                                      ----------                              ----------------------------------------------
                        Min or Max  2.900                                 -2.894   4.7      138.00      3.678  53.00

[  1645] = 1+X,-1+Y,Z
                                                                      "6ORC     "  PLATON-GEOMETRY  Page   48
=====================================================================================================

Analysis of Y-X...Cg(Pi-Ring) Interactions (X..Cg < 4.0 Ang. - Gamma <  30.0 Deg)
=====================================================================================================

  Y--X(I)   Res(I)  Cg(J)  [   ARU(J)]     X..Cg  Transformed J-Plane P, Q, R, S  X-Perp Gamma    Y-X..Cg      Y..Cg Y-X,Pi

C2    -S2    [ 1] -> Cg1   [  2566.01]  3.4689(19)  -0.8291-0.5586-0.0240  -3.7346 -3.425   9.13   89.60(13)    3.835(4)   0.53
C12   -S12   [ 2] -> Cg2   [  2656.02]    3.619(2)  -0.9059-0.4207-0.0485  -3.5236 -3.530  12.77   92.94(14)    4.066(4)   1.21
C14   -O14   [ 2] -> Cg1   [  2666.01]    3.385(4)  -0.8291-0.5586-0.0240 -10.2729  3.192  19.43     78.7(2)    3.368(4)   8.09
                                      ----------                              ----------------------------------------------
                        Min or Max  3.385                                 -3.530   9.1      92.94      3.368   8.09

[  2566] = -X,1-Y,1-Z
[  2656] = 1-X,-Y,1-Z
[  2666] = 1-X,1-Y,1-Z
```

Table S3 (cont.). π-stacking interactions analysis of PTU cocrystals.

PTU-HQ

6-Membered Ring (1) N1 --> C2 --> N3 --> C4 --> C5 --> C6 -->
6-Membered Ring (2) C11 --> C12 --> C13 --> C11_a --> C12_a --> C13_a -->

Analysis of Short Ring-Interactions with Cg-Cg Distances < 6.0 Ang., Alpha < 20.000 Deg. and Beta < 60.0 Deg.

Cg(I)	Res(I)		Cg(J)	[ARU(J)]	Cg-Cg	Transformed J-Plane P, Q, R, S	Alpha	Beta	Gamma	CgI_Perp	CgJ_Perp	Slippage
Cg1	[1]	->	Cg1	[1455.01]	5.777(3)	0.6051-0.4845 0.6317 1.9437	0.00(8)	52.8	52.8	3.4959(7)	3.4959(7)	4.599
Cg1	[1]	->	Cg1	[1655.01]	5.777(3)	0.6051-0.4845 0.6317 8.9355	0.00(8)	52.8	52.8	3.4959(7)	3.4959(7)	4.599
Cg1	[1]	->	Cg1	[2666.01]	4.207(2)	-0.6051 0.4845-0.6317 -1.8439	0.00(8)	31.3	31.3	3.5957(7)	3.5957(7)	2.184
Cg1	[1]	->	Cg2	[1655.02]	5.355(3)	0.4052 0.9101 0.0873 4.5658	81.92(8)	17.6	84.2	0.5436(7)	5.1057(7)	
Cg1	[1]	->	Cg2	[2657.02]	5.355(3)	-0.4052-0.9101-0.0873 -4.5658	81.92(8)	17.6	84.2	0.5436(7)	5.1057(7)	
				Min or Max	4.207		0.0	17.6	84.2	-3.596	-3.596	

[1455] = -1+X,Y,Z
[1655] = 1+X,Y,Z
[2666] = 1-X,1-Y,1-Z
[1655] = 1+X,Y,Z
[2657] = 1-X,-Y,2-Z

Table S4. Maximum apparent solubility (S_{max}) of PTU and its cocrystals in pH 6.8 phosphate buffer medium.

	S_{max} (mg·mL^{-1})	Time to reach S_{max} (min)	Solubility enhancement
PTU	1.28	30	
PTU-CAT	1.26	5	0.98X
PTU-RES	1.69	10	1.32X
PTU-ORC	1.66	30	1.29X
PTU-HQ	1.67	5	1.30X
